# Touching with torque enables human-level robotic dexterity

**DOI:** 10.1126/sciadv.aec3263

**Published:** 2026-03-04

**Authors:** Ling Wang, Yu Sun, Laihao Yang, Yuxuan Sun, Qingkai Guo, Yixue Liu, Xuefeng Chen, Yajing Shen

**Affiliations:** ^1^ School of Mechanical Engineering, Xi’an Jiaotong University, Xi’an 710049, China.; ^2^ HKUST Shenzhen Research Institute, Shenzhen 518057, China.; ^3^ Department of Electronic and Computer Engineering, The Hong Kong University of Science and Technology, Hong Kong SAR, China.; ^4^ Center for Smarting Manufacturing, The Hong Kong University of Science and Technology, Hong Kong SAR, China.

## Abstract

Achieving human-like forceful manipulation remains a major challenge in robotics because of the lack of critical environmental interaction cues such as collisions, balance, and resistance. We present a torque-angle-pressure (TAP) tactile sensor leveraging magnetic flux density gradients to achieve bidirectional, ultrasensitive (~0.1°, ~0.4 newton-millimeter), and high-linearity (*R*^2^ = 0.99) sensing over a wide range (±241.6 newton-millimeter) through a single readout channel. The accurate torque sensing ability provides both force and distance information, bringing the environment into the interaction loop. A TAP-equipped robot can perform vision-free stable object placement and complete a balance beam stacking challenge in just 2.4 seconds with a success rate of 81.5%—both measured metrics surpassing human performance. It also supports adaptive daikon slicing with real-time posture and motion adjustments—capabilities rarely achievable in existing robotic systems. This work advances tactile sensing, enables forceful manipulation in unstructured environments, and represents a key step toward effective human-robot collaboration.

## Teaser

A TAP sensor enables vision-free forceful manipulation, surpassing human performance via ultrasensitive magnetic sensing.

## INTRODUCTION

**Fig. 1.**
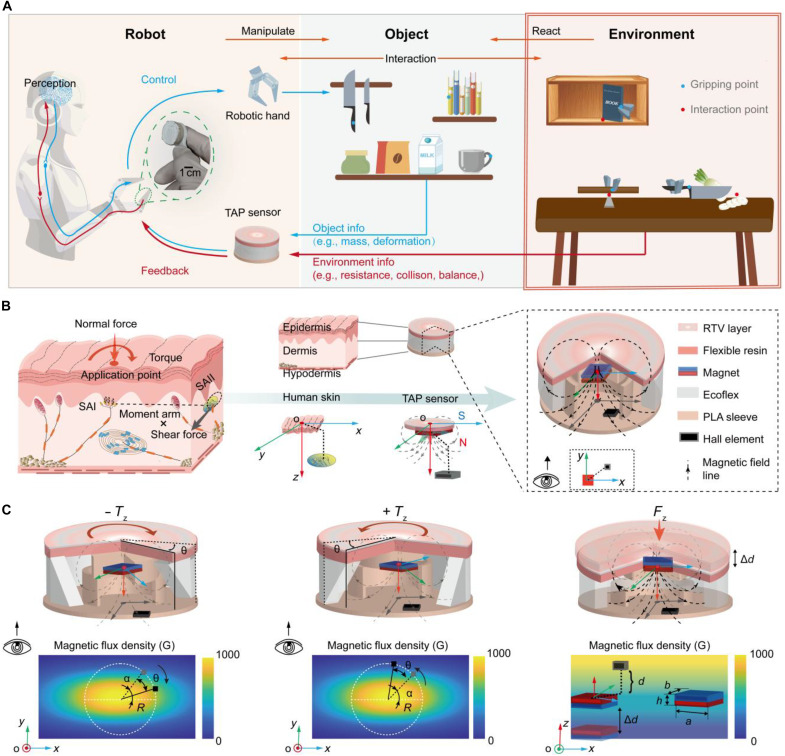
Overview of the interaction-in-the-loop framework with the TAP sensor that integrates interaction information with the environment during forceful manipulation. (**A**) Robots achieve interaction information with the environment by processing tactile information, such as pressure, torsion angle, and torque, enabling various forceful manipulation tasks, including precise object placement, beam balancing, and vegetable slicing. (**B**) Skin-inspired design of the TAP tactile sensor. Left: Distribution and sensation characteristics of tactile mechanoreceptors beneath the human skin. SAIs are sensitive to pressure distribution. SAIIs are sensitive to shear force. Middle: Mimicking the spatial distribution of tactile corpuscles SAIs. The Hall element and magnet are offset in space. Right: Monolithic structure of the tactile sensor. (**C**) Mechanism of the TAP sensor to detect compressive deformation and bidirectional torsion. Left: Under counterclockwise torque, the Hall element revolves counterclockwise around the magnet’s center at a fixed radius. Middle: Under clockwise torque, the Hall element revolves clockwise around the magnet’s center at a fixed radius. Right: Under pressure, the magnet and Hall element move closer to each other.

Achieving human-like dexterity in robotics is crucial for automating precise tasks across various fields, including industry, health care, and daily life. Despite recent advancements that have substantially improved robotic grasping and holding capabilities, replicating the nuanced forceful manipulation skills of humans remains a formidable challenge (*1*–*8*). One major issue is that current research primarily focuses on manipulating the object itself, frequently overlooking the environmental reaction resulting from inadequate contact sensing (Fig. 1A). While some approaches use artificial intelligence–enhanced vision systems to estimate object changes during manipulation (*9*–*11*), they often struggle with real-time physical interactions such as collisions, balance, and resistance, like placing a carton of milk on a table or slicing vegetables. Accurately capturing this interaction—especially during direct physical contact with the environment—remains difficult, hindering robots from performing simple, essential tasks that humans accomplish effortlessly (*12*).

The spatially anisotropic arrangement of tactile receptors in human fingertips allows for the accurate perception of force, position, and stretching, with the integrated processing of these signals enabling precise torque perception during manipulation (*13*–*18*). This integration of force and position is vital for tasks involving off-center manipulation, where the positional correlation between the contact point and the applied force directly affects stability and control. In robotics, however, replicating this level of integration remains a substantial challenge. Although recent advancements in tactile sensors have introduced various mechanisms for contact force measurement, they often fail to capture critical positional information—specifically, the spatial correlation between the clamping point and the point of external force application (*19*, *20*). This limitation often leads to failures in off-center manipulation tasks, which are common in practice, even when closed-loop control is used (*21*–*23*).

Soft tactile sensors using electrical signals (e.g., piezoresistive, capacitive, triboelectric, etc.) with three-dimensional (3D) structure arrays (e.g., pyramid-plug, tenon-and-mortise, origami, and cilia) can measure torque or torsion angles on the basis of signal differences from multiple elements (*6*, *14*, *24*–*35*). However, they often struggle to achieve a high resolution, wide range, linearity, and bidirectional capability because of low shear force resolution and signal coupling. Vision-based optical sensors can detect torsion changes through slip measurements but lack quantitative torque data. Extending the torque measurement range by converting surface deformation into marker displacement also faces issues like low linearity (<0.9), poor resolution (>2 N·mm), and slow response times resulting from complex material deformation. Similarly, magnetic field–based sensors cannot achieve torsion measurement when arranged centrally, while those arranged in an array configuration only indicate torsion trends without providing precise torque values (*36*, *37*). Therefore, developing tactile sensors capable of detecting torque with high sensitivity, wide range, high linearity, and bidirectional capability is essential for achieving human-like dexterity and adaptive manipulation.

This paper introduces a torque-angle-pressure (TAP) tactile sensor inspired by the spatial distribution of human tactile corpuscles (Fig. 1B). The TAP sensor leverages magnetic flux density gradients to achieve ultrasensitive, wide-range, and bidirectional torque sensing. It detects minute torsion changes (~0.1°) and torque variations (~0.4 N·mm) with a high linearity (0.99) across an expansive range (241.6 N·mm) while maintaining bidirectional measurement capability with a single readout, significantly outperforming existing techniques (table S1). The TAP sensor directly integrates quantitative environmental feedback into the robot interaction loop, implementing an interaction-in-the-loop framework. This approach allows robots to dynamically adjust posture and actions in real time, enabling previously unattainable precise forceful manipulation, including stable object placement on shelves without visual guidance, balance beam stacking, and adaptive vegetable slicing. Compared to existing techniques, the TAP sensor not only achieves superior resolution, range, and linearity but also uniquely integrates force and spatial information within the interaction loop. This capability greatly enhances robot dexterity in unstructured environments, advancing human-robot collaboration.

## RESULTS

### TAP sensor design, fabrication, and working principle

Human tactile mechanoreceptors beneath the skin detect both pressure (SAI) and shear forces (SAII). Furthermore, tactile corpuscles are also capable of synergistically perceiving both radial and circumferential skin stretching to identify more complex load states (e.g., slippage and torsion) (*18*, *38*). Inspired by the hierarchical structure of the human skin and the asymmetric spatial distribution of tactile corpuscles, we designed a TAP tactile sensor that can simultaneously detect torsion angle, torque, and pressure through a layered structure (Fig. 1B, middle and right).

The TAP sensor consists of a three-layer sandwich structure designed to mimic the tactile functionality of the human skin. The top layer features a flexible resin cap (Flexible 80A Resin, 8.9 MPa) coated with RTV (room-temperature vulcanizing silicone) and embedded with a rectangular magnet (N35, Br ~ 1200 mT). This layer replicates the epidermal response to tactile stimuli while stabilizing the contact interface under torque (fig. S1). The middle layer, made of an annular elastomer (Ecoflex 00-50, *E* ~ 81 kPa, *G* ~ 27 kPa), functions as a force transmission medium similar to the soft dermis, and its mechanical mismatch with the top layer enhances force sensitivity. The bottom layer, an interlocking sleeve frame of polylactic acid (PLA) containing a Hall element, is responsible for detecting magnetic field variations, effectively resisting shear force coupling.

The TAP sensor design carefully considers the spatial arrangement of the magnet and the Hall element to achieve accurate torque and pressure measurement (Fig. 1B). A rectangular magnet characterized by dimensions (*a*, *b*, and *h*), magnetized along its thickness, generates an anisotropic magnetic field essential for precise torque detection. Compared to a triangular or hexagonal magnet, the circumferential magnetic flux density induced by an easily manufactured rectangular magnet demonstrates a broader monotonic range and a more pronounced gradient, which contribute to optimizing sensor performance (linearity, sensitivity, and range) (fig. S2). The Hall element, positioned at an optimized spatial offset from the magnet, characterized by parameters *R* (horizontal fixed radius), *d* (normal distance), and α (initial angle), detects localized magnetic flux density variations as the sensor deforms. This spatial configuration enhances the sensor’s response to multidimensional tactile stimuli while minimizing interference from tangential forces (text S2).

During torque application, the Hall element revolves in-plane around the magnet at a fixed radius *R* from the initial angle α, capturing circumferential displacement (Fig. 1C, left and middle). This rotational motion reflects changes in the circumferential magnetic flux density, allowing accurate detection of torsion. When pressure is applied, the Hall element moves closer to the magnet, capturing changes in normal displacement (Fig. 1C, right). This dual-motion sensing is crucial for distinguishing torsion from pressure, allowing the sensor to detect both types of deformation accurately.

### Theoretical basis and design optimization

The relationship between the Hall element’s output voltage and measurement parameters (normal displacement and torsion angle) is theoretically derived on the basis of Maxwell’s electromagnetic field theory and the Hall effect principle (text S2). Specifically, it is worth noting that to decode the signal obtained from anisotropic sensor architecture, which exhibits evident nonlinearity in all three dimensions, a new theoretical model relating the magnetic field *B*_z_ to the radial coordinate *R* and the circumferential angle θ was established (text S2). Only with this model can the torsion angle be accurately determined by ${\left.\frac{\partial B_{z}}{\partial \theta }\right|}_{R=R_{0}}$. According to the Hall effect, the final voltage output is linked to the average magnetic flux density along the *z* axis in the plane of the Hall element, expressed as

$$\Delta U=K_{H}B_{\text{Hall}}\;\left(R\text{cos}\;\theta_{B},R\text{sin}\;\theta_{B},z\right)$$ (1)

where $K_{H}$ is the electrical parameter of the Hall element, valued at −1.708 × 10^−3^, and $B_{\text{Hall}}$ is the average magnetic flux density along the *z* axis in the plane of the Hall element. Combining this theoretical model with experimental calibration enables the TAP sensor to sequentially measure normal displacement (Δ*z*) and the torsion angle (Δθ_B_). The torque-torsion angle and pressure-normal displacement relationships follow Hooke’s law

$$P={\mathrm{ES}}_{e}\Delta z/L_{e}$$ (2)

$$T_{z}=GJ\Delta \theta_{B}/L_{e}$$ (3)

where *E* and *G* denote the elastomer’s elasticity and shear modulus, respectively; *S*_e_ and *J* represent the axes and polar moment of inertia; and $L_{e}$ is the elastomer height.

**Fig. 2.**
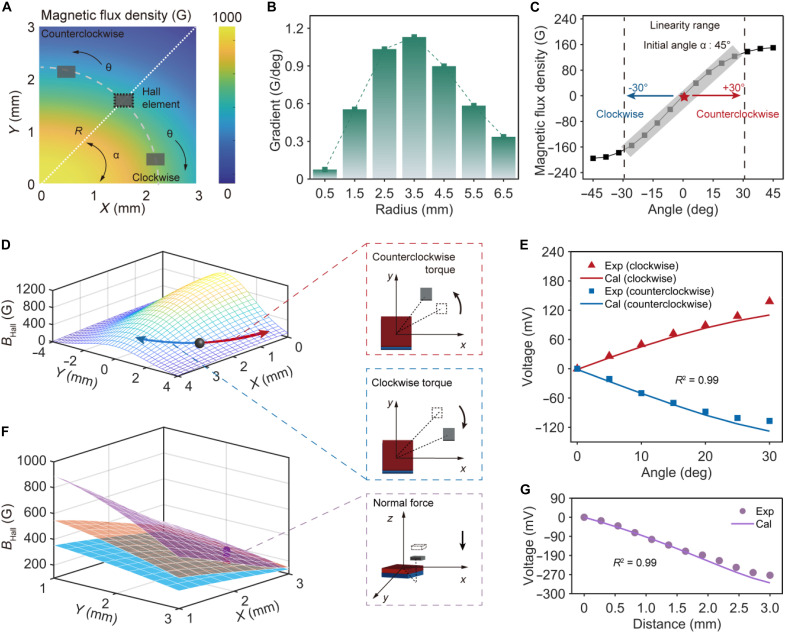
Torque-torsion-angle sensing mechanism and validation. (**A**) Schematic diagram of the torsion sensing mechanism for the TAP sensor. The Hall element can detect the variation of magnetic flux density when it revolves in an anisotropic magnetic field generated by the rectangular magnet, enabling torsion measurement. (**B**) Demonstrations of magnetic flux density gradient variation when the radius *R* changes from 0.5 to 6.5 mm, indicating torsion sensing sensitivity variation. (**C**) Illustrations of the magnetic flux density detected by the Hall element when it revolves from −45° to 45°, showcasing high linearity in the range from −30° to 30°. (**D**) Magnetic flux density distribution along the circumferential direction generated by the rectangular magnet. The magnetic flux density shows a bidirectional variation tendency along two opposite revolving directions. (**E**) Theoretical and experimental responses of the TAP sensor to torsion stimuli in opposite directions. The well-matched results between the theoretical and experimental data achieve a high fit rate of 0.99, demonstrating the validity of the theoretical analysis. (**F**) Magnetic flux density distribution along the normal direction generated by the rectangular magnet. The magnetic flux density decreases with the locations far from the center and with increasing distance *d*. (**G**) Theoretical prediction and experimental measurement of the TAP sensor when applying pressure stimuli in the normal direction. The fit rate between the theoretical and experimental data approaches 0.99, presenting a well-matched tendency and thus further convincing the theoretical results.

To achieve a balance between sensitivity and avoiding saturation, the initial normal distance (*d*) is set to 5 mm, with a maximum normal displacement range Δ*d* = 3 mm (figs. S7 to S9). To further improve torsion measurement accuracy, the circumferential magnetic flux density gradient was optimized. As shown in Fig. 2A, adjusting the magnetic flux density gradient significantly enhances torsion sensing performance. Experimental comparisons indicate that a magnet with dimensions (*a* = 6 mm and *b* = 3 mm) offers the best balance between gradient and linear range, while increasing the magnet height (*h* = 2 mm) further improves resolution (fig. S10). This combination enables the sensor to maintain precise torsion detection even under variable loading conditions.

To fine-tune the torsion sensing performance, spatial offset parameters were carefully selected: *R* is set to 3.5 mm, achieving a torsion angle resolution of 0.1° (Fig. 2B and figs. S11 and S12). For bidirectional torsion perception, the initial angle (α) is set to 45°, leveraging the symmetrical property of the circumferential magnetic flux density (Fig. 2C). This spatial arrangement ensures optimal sensitivity to minute torsion changes while maintaining a high linearity (0.99) over an effective measurement range of 15° to 75° (Fig. 2, D to G, and fig. S13). In addition, selecting an elastomer with a higher modulus (Ecoflex 00-50) significantly enhances torque detection, allowing for wide-range torque measurement. These results align with theoretical calculations, confirming the sensor’s effectiveness in precise torque and pressure sensing. Overall, we introduce the conceptual framework and spatial offset design in the presence of an anisotropic magnetic field gradient. Additional details on the optimization design of the TAP sensor can be found in texts S3 and S4.

### Superior performance of TAP sensor in linearity, resolution, range, and bidirectional sensing

**Fig. 3.**
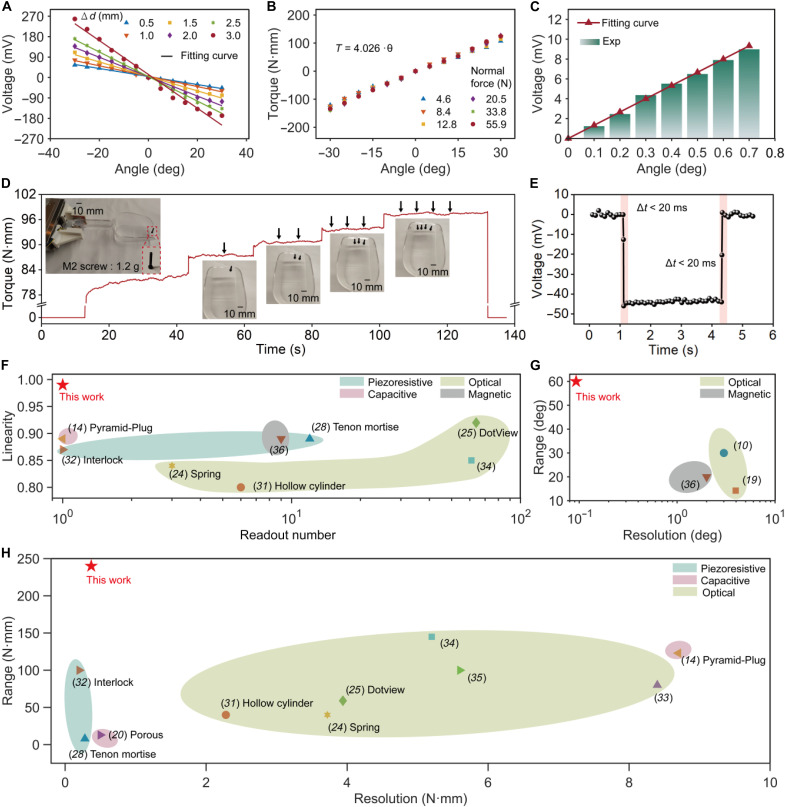
TAP sensor performance calibration. (**A**) Voltage output of the TAP sensor when applying torsion angle changes from −30° to 30° under different initial compression deformations varying among 0.5, 1.0, 1.5, 2.0, 2.5, and 3.0 mm, indicating high robustness to maintain superior linearity without being affected by the initial pressure conditions. (**B**) Relationship between torque and torsion angle under different pressures of 4.6, 8.4, 12.8, 20.5, 33.8, and 55.9 N. (**C**) Demonstrations of the Hall element’s voltage response to incremental torsion angle changes of 0.1°, showcasing the ultrahigh resolution of the TAP sensor for torsion angle. (**D**) Illustrations of the TAP sensor to detect wideband and tiny torques by grasping a heavy ice scoop while sequentially adding four small screws. (**E**) Response time and recovery time of the TAP sensor. (**F** to **H**) Comparison of previously reported piezoresistive, capacitive, magnetic, and optical measurement principles with our TAP sensor in terms of range, resolution, linearity, and readout number. Shaded and dashed circle groups are related references based on the same principle. For unlabeled references, the sensors are all planar multilayer structures (see table S1 for details).

To characterize the TAP sensor’s performance, commercial pressure and torque sensors were used for calibration (fig. S14). As shown in Fig. 3A, the sensor demonstrates excellent linearity (up to 0.99) within a wideband measurement range (±30°) under all pre-pressure conditions. Notably, the sensor exhibits a higher resolution under increased external pressure. For instance, the resolution under the maximum pressure state (Δ*d* = 3 mm, 55.9 N) is 4.21 times higher than that under the initial state (Δ*d* = 0.5 mm, 4.6 N). This capability is particularly beneficial for robotic manipulation as it ensures stable and accurate torque sensing even when the gripper applies a significant holding force. In addition, the circular elastomer design ensures consistent linearity regardless of pre-pressure, with a calibrated torque-to-torsion angle coefficient of 4.03, resulting in a torque measurement range of ±120.9 N·mm (total: 241.8 N·mm) (Fig. 3B). The sensor also exhibits consistent linearity and range in both directions, confirming its bidirectional torsion sensing capability. Overall, this is a soft torque sensor capable of bidirectional torsion sensing with a single readout, significantly simplifying signal acquisition and reducing calibration time.

The TAP sensor’s exceptional resolution is further demonstrated in Fig. 3C, where the Hall element’s voltage response to incremental torsion angle changes of 0.1° shows a clear, linear voltage step of 1 mV (movie S1). This resolution surpasses that of existing sensors by more than an order of magnitude (*36*). Calibrated using the torsion-torque relationship (Eq. 3), the torque resolution reaches 0.4 N·mm. In addition, a random dynamic loading test confirms the sensor’s high dynamic measurement accuracy, with a normalized root mean square error of 2.37% (fig. S15). Moreover, the TAP sensor also demonstrates robust environmental adaptability, with its output remaining virtually unchanged across temperatures ranging from 25.5° to 79.9°C and humidity levels from 16 to 82% (fig. S16). It has been demonstrated that the sensor’s output fluctuation is a mere 0.09% when subjected to a stray magnetic field of ~1.14 mT (fig. S17). In addition, the sensor displays stability in performance following 10,000 cycles of loading (fig. S18). We further conducted a parallel comparison of sensors from different batches, and the results demonstrated high consistency across sensors, indicating good cross-batch reproducibility (fig. S19).

To validate the sensor’s practical capability, a holding task was conducted (Fig. 3D). A gripper equipped with the TAP sensor grasped an ice scoop (96 g, initial torque of 82 N·mm) while four screws (1.2 g each) were sequentially added. Despite significant preapplied loads, the sensor accurately detected each incremental torque change of ~2.4 N·mm per screw, demonstrating its high sensitivity. Moreover, the fast response time (<20 ms) highlights its suitability for dynamic manipulation tasks (Fig. 3E and fig. S20).

A comparative analysis (Fig. 3, F to H, and table S1) demonstrates that the TAP sensor significantly outperforms conventional tactile sensors in resolution, linearity, range, and readout efficiency. Unlike existing soft tactile sensors, the TAP sensor uniquely integrates torsion angle and torque measurement within a single readout system, achieving an unmatched resolution (0.1° for torsion and 0.4 N·mm for torque), expansive range (up to 241.8 N·mm), and high linearity (0.99). It also achieves wide-range pressure sensing (up to 55.9 N), effectively doubling the measurement range compared to conventional designs. Notably, the torsion angle resolution is improved by 20-fold, while the torque resolution is enhanced by an order of magnitude. These capabilities enable interaction sensing between objects and their environment. By incorporating this feedback into the robot control loop, the TAP sensor serves as a key enabler for dexterous manipulation and human-robot collaboration, advancing the robot’s intelligence.

### Interaction-in-the-loop framework and ultraprecise placement tasks

Precisely picking and placing objects in unstructured environments are crucial for robots across various applications, such as logistics and domestic services (*39*–*41*). While picking tasks are relatively straightforward, precise placement remains challenging—especially in visually restricted scenarios—because of the complex robot-environment interactions, including collisions, balance, and resistance. Even with vision feedback, achieving stable placement is difficult as the robot must continuously adjust to prevent tipping. Conventional methods relying solely on force or position information often fail to fully incorporate the robot-environment interaction, making ultraprecise placement tasks particularly difficult.

**Fig. 4.**
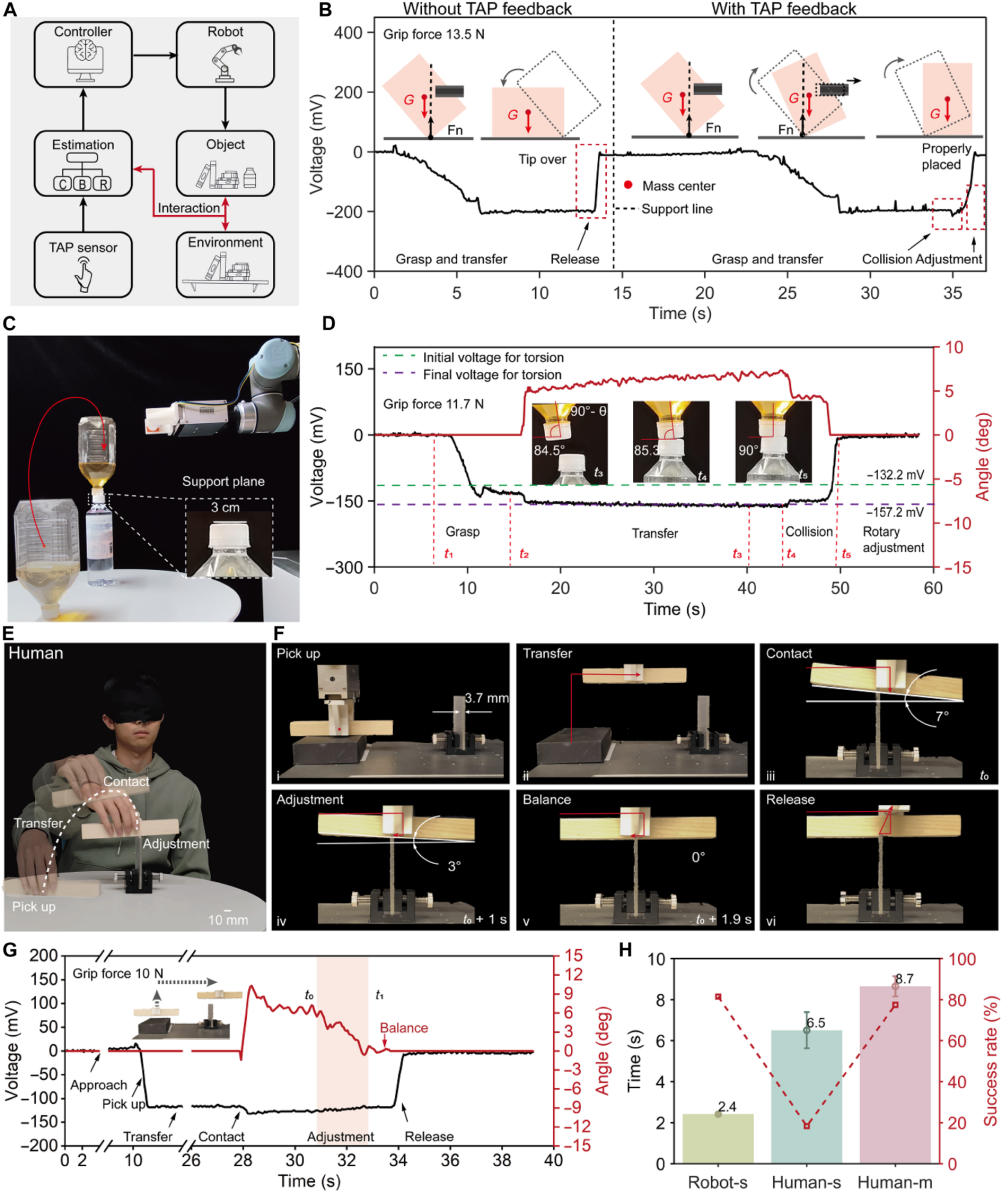
Ultra-precise placement tasks. (**A**) Schematic diagram of the interaction-in-the-loop framework that integrates interaction information (e.g., collisions, balance, and resistance) into the robot control loop through the ultrasensitive and wideband TAP sensor. (**B**) Comparison of tasks of directly placing the milk carton at an inclined angle of 46° and completing it with the interaction-in-the-loop framework. The latter experiment, facilitated with the TAP sensor feedback, succeeded, while another one failed without TAP sensor feedback. (**C**) Placement task of a larger bottle (half-filled with water) upside down on the cap of a smaller bottle. (**D**) Variation of the torsion angle during the placement process. The inverted bottle is grasped with an initial twist angle of 5.5°, and then the placement task is stably completed through elaborate adjustments with real-time torsion feedback. (**E**) The beam balancing experiment was conducted by volunteers without vision guidance. Twelve volunteers were invited to complete this test. (**F**) Beam balancing experiment of the robot with the torsion feedback through the TAP sensor. (**G**) Variation of torsion information during the beam balancing process, including picking up, transferring, collision detection, balance detection, and releasing. (**H**) Comparison of the time and success rate for the robot and human to stack a balance beam. Robot-s represents a single adjustment for the robot. Human-s denotes a single adjustment for humans. Human-m indicates multiple adjustments for humans. The error bars represent the standard error (SE).

To address these challenges, we propose an interaction-in-the-loop framework, leveraging the ultrasensitive and wideband TAP sensor. As shown in Fig. 4A, this framework continuously integrates interaction information (e.g., collisions, balance, and resistance) into the robot control loop, aiming to complete tasks through real-time assessment of these interactive states. The controller adopted in this framework implements a hybrid force-position control method. By incorporating real-time interaction state feedback, the robot can dynamically adjust posture and action, allowing precise placement in unstructured environments. To implement this framework, we developed an end-effector equipped with the TAP sensor, mounted on a gripper to detect critical interactions. The following experiments showcase the framework in increasingly complex placement tasks: placing a packaging box, balancing a large bottle on a smaller one, and performing a beam balancing challenge.

In the first scenario, the TAP sensor–equipped gripper grasps a packaging box (weight: 214 g; dimensions: 206 mm by 106 mm by 35 mm) and places it stably onto a shelf (figs. S21 and S22). To highlight its adaptivity in unstructured environments, we introduce an inclined milk carton (inclined angle of 46°) to the robot. In the initial state, the carton would typically fall after placement. However, guided by the TAP sensor guidance, the robot performs real-time detection of contact orientation, adaptive tracking of gravity center shifts, and closed-loop optimization of the grasping angle, demonstrating the system’s robustness and the effectiveness of the interaction-in-the-loop framework (Fig. 4B and movie S2). Leveraging real-time torsion angle feedback, the robot continuously adjusts the contact angle to an extremely small value, achieving stable placement within 4 s, effectively minimizing tipping risk. Additional placement demonstrations with various everyday objects (e.g., soap, cups, and shampoo bottles) further showcase its adaptability for diverse interaction surfaces (movie S2).

The second task involves placing a larger bottle (half-filled with water) upside down on the cap of a smaller bottle. This challenge is particularly demanding because of the convex curvature of the cap (area <7 cm^2^) and the dynamic shift of liquid as the bottle tilts (Fig. 4C). During grasping, an initial twist (θ ≈ 5.5°) occurs as a result of the offset between the grasp point and the bottle’s center of gravity (Fig. 4D and movie S3). The TAP sensor accurately detects this initial torsion, allowing the robot to fine-tune the bottle’s orientation. Upon contact with the smaller bottle, the robot adjusts the twist angle from 5.5° to 4.7° (at time *t*_4_) and then to near 0° (at time *t*_5_), completing the stable upside-down placement in under 5 s.

The final scenario tackles a highly challenging task—even for humans: balancing a beam (140 mm by 20 mm) on a thin board (thickness: 3.7 mm). This task requires ultraprecise torsion sensing and real-time feedback to adjust the beam’s orientation dynamically. Using the multistep control strategy with real-time bidirectional torsion feedback, the gripper achieves stable beam placement in just 1.9 s (Fig. 4, F and G, and movie. S4). To verify the robustness, the experiment was repeated 27 times with varying collision points between the beam and the board. We further benchmarked the robotic system against human performance by testing 12 volunteers on the same task under visual deprivation using single-adjustment and multiple-adjustment strategies (figs. S23 and S24). As shown in Fig. 4H, the average completion times for Robot-s, Human-s, and Human-m are 2.4, 6.5, and 8.7 s, with corresponding success rates of 81.5, 18.3, and 77.5%, respectively. The respective 95% confidence intervals were [2.3 s, 2.5 s], [4.7 s, 8.3 s], and [7.7 s, 9.6 s] (fig. S25A). To further assess performance across the three groups, the task completion time can also be normalized using *T*e, a metric that incorporates both the time of successful trials and the overall success rate (fig. S25B). In conclusion, the robot, operating via tactile feedback, significantly outperformed all human trials, attaining the shortest completion time (2.4 s) and the highest success rate (81.5%). This substantial performance highlights the TAP sensor’s ability to handle complex placement tasks with high precision and efficiency. Furthermore, we compared the visual guidance–based placement task, which is still hard to complete because of lighting conditions, working distance (>500 mm), and the narrow dimensions or small size of the target object (<4 mm) (fig. S26 and table S4). Although it might be attainable with higher-precision cameras (e.g., structured light camera) and stricter lighting controls, the setup would be very sophisticated. Our approach, the TAP sensor, provides a direct and intuitive way for this kind of task by detecting minute torsion/torque.

In summary, the TAP sensor’s real-time torsion sensing and interaction-in-the-loop framework enable robots to perform ultraprecise placement tasks, even in unstructured environments. By effectively addressing challenges such as collisions, balance, and resistance, the TAP sensor–equipped robot demonstrates a significant improvement over conventional systems, achieving human-like precision and stability.

### Interaction-in-the-loop forceful manipulation: Slicing vegetables

**Fig. 5.**
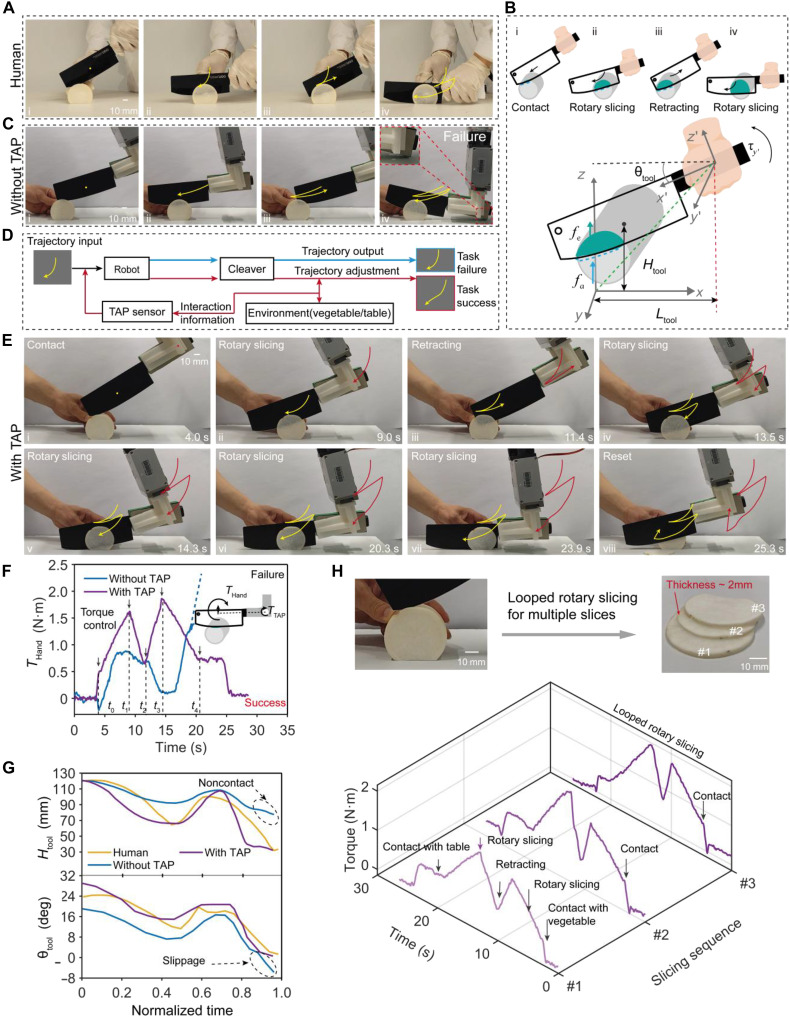
Vegetables slicing tasks. (**A**) Illustration of the looped rotary slicing strategy for daikons. The process involves multiple repeated steps: contact, rotary slicing, retracting, and rotary slicing. (**B**) Schematic diagram of the mechanical model for the rotary slicing strategy. (**C**) Demonstration of conventional methods replicating the looped rotary slicing trajectory in an attempt to slice the daikon solely relying on planned movements. It fails to make sense without TAP sensor feedback. (**D**) Replication of the looped rotary slicing trajectory by integrating the interaction-in-the-loop framework through TAP sensor feedback. (**E**) Daikon slicing task using the looped rotary slicing strategy through TAP sensor feedback. The robot can dynamically adapt the slicing motion and succeed in this task. (**F**) Comparison of torque variation during looped rotary slicing trajectory replication with and without TAP sensor feedback. (**G**) Variation of the slicing parameters $H_{\text{tool}}$ and $\theta_{\text{tool}}$ obtained by using different slicing strategies. (**H**) Torque variation during continuous-slicing tasks with TAP sensor feedback to adjust the slicing motion.

Slicing vegetables, especially tough varieties like daikon, presents a substantial challenge for robots, as they must continuously adjust their cutting strategy on the basis of real-time interaction feedback between the knife and the vegetable. Humans naturally adopt a rotary slicing strategy to maintain balance and reduce resistance compared to a direct up-down cutting motion (Fig. 5A, fig. S27, and movie S5). This strategy leverages the tool’s inclined angle and the distance between the cleaver’s center and the object, dynamically adjusting the cutting force to maintain stability (*42*).

To better understand the rotary slicing strategy, we developed a mechanical model to analyze the interaction between the cleaver and the vegetable (Fig. 5B). In this model, θ_tool_ represents the tool incline angle, $L_{\text{tool}}$ is the distance between the cleaver’s center and the object’s center, $H_{\text{tool}}$ is the distance between the cleaver’s center and the table, $f_{a}$ denotes the fracture force, and $f_{e}$ represents the frictional resistance between the cleaver and the fracture surface. The model reveals that reducing the tool incline angle (θ_tool_) and maintaining a stable distance ($L_{\text{tool}}$) can effectively minimize resistance, enhancing cutting stability. Humans intuitively adapt these parameters during slicing, allowing them to maintain balance and efficiency (fig. S28).

Traditional robotic slicing methods that mimic human hand trajectories often fail, as shown in Fig. 5C. These methods overlook the fundamental differences in mechanical characteristics between human hands and robotic end-effectors. While humans can simultaneously sense torque and adaptively control the cutting path, robots lack this ability. In practice, the cutting resistance varies significantly between different vegetables, making it unreliable for robots to follow a predefined trajectory without real-time adjustment. Consequently, forceful slicing tasks become inefficient and prone to failure when solely relying on planned movements.

To address these challenges, we propose an interaction-in-the-loop slicing strategy that leverages the TAP sensor’s real-time torque feedback to dynamically adapt the slicing motion. The TAP sensor precisely measures torque ($\tau_{y^{\prime }}$), thereby estimating the interaction force ($f_{a}$ and $f_{e}$) and relative acting point. This capability integrates environmental information into the control loop, enabling the robot to adjust the end-effector posture and output in real time, ensuring stable and successful slicing (Fig. 5D). To demonstrate this strategy, we developed an end-effector gripper that integrates a TAP sensor at the trailing end to detect torque and an elastomer pad at the leading end to extend the measurement range (figs. S29 and S30). In the cucumber slicing task (fig. S31), the rotary slicing strategy, guided by real-time torque feedback from the TAP sensor, enabled the robotic hand to perform smooth and stable one-step slicing. The cleaver, mounted on the robotic hand, effortlessly cut through the cucumber without tipping or excessive resistance, demonstrating the effectiveness of the proposed slicing strategy.

To further validate the rotary slicing strategy, we conducted experiments with daikon, a tougher and larger vegetable requiring a looped rotary slicing strategy. This multistep approach involves contact, rotary slicing, and retracting, efficiently handling larger dimensions and harder textures. During the experiment, we calibrated the torque *T*_hand_ through the output of the TAP sensor (fig. S30). The *T*_hand_ detected the initial contact between the cleaver and daikon at $t_{0}$, with a torque of 0.45 N·m. As rotary slicing progressed ($t_{0}$-$t_{1}$), the torque increased to 1.6 N·m at $t_{1}$, indicating high resistance (Fig. 5, E and F). Consequently, the robot temporarily halted and retracted until the torque decreased to 0.6 N·m at $t_{2}$. The process was repeated with a final torque threshold of 1.8 N·m, ensuring complete severance ($t_{2}$-$t_{3}$).To enhance stability during the final cut, the robot adjusted the cleaver to be nearly parallel to the working surface while maintaining sufficient contact force ($t_{3}$-$t_{4}$). The entire slicing task was completed in 20 s, demonstrating the precise control of the cutting force. This final adjustment ensured a complete and stable separation, as depicted in movie S5.

Figure 5 (F and G) further compares the motion trajectory extracted from the motion of the cleaver during the slicing process (fig. S32). When using the rotary slicing strategy integrated with TAP sensor feedback, the robotic hand attitude can be precisely adjusted in real time when subjected to the contact and resistance induced by the target daikon-cleaver interaction (Fig. 5G and movie S5). Thus, the robot hand perfectly follows the extracted trajectory during the slicing process. In contrast, the cutting strategy without the TAP sensor feedback often led to disturbances resulting from excessive resistance, causing the cleaver to deviate from the intended trajectory severely (Fig. 5G).

In addition, by embedding or identifying the dimensions of the target daikon, automatic continuous-slicing tasks can be implemented on the basis of the looped rotary slicing strategy with TAP sensor feedback. The experiment for the continuous-slicing task to obtain multiple slices is showcased in Fig. 5H, fig. S33, and movie S5. By reducing the lateral stepping value of the robotic arm, thinner slices can be achieved, almost down to 1.2 mm (fig. S34). These results demonstrate the TAP sensor’s capability to bring environmental information into the control loop, enabling real-time adjustment and adaptive forceful manipulation during slicing, which is crucial for enhancing robotic cutting performance.

## DISCUSSION

Achieving nuanced forceful manipulation skills comparable to humans is essential for automating precise tasks across various sectors. This study addresses a critical challenge in robotics: integrating interaction information with the environment during forceful manipulation. Traditional approaches often overlook the importance of torsion angle measurement and torque feedback control, limiting robots’ ability to interact with unstructured environments. To overcome this limitation, we present an interaction-in-the-loop framework that leverages the TAP sensor’s ultrasensitive and wideband torque detection to continuously monitor key robot-environment interactions. This framework enables robots to accurately sense forces and relative positioning in real time, facilitating precise adjustments during manipulation. The proposed method substantially enhances the robot’s capability to perform forceful tasks, particularly in complex, unstructured environments, demonstrating potential applications in various fields, including industrial automation and service robotics.

This study establishes a TAP measurement principle on the basis of the spatial arrangement of the magnet and the Hall element. By theoretically modeling the Hall element’s output voltage for any position within the magnetic field generated by a rectangular magnet, we introduce a method to precisely measure torque and torsion angle through engineered spatial offset design in the presence of an anisotropic magnetic field gradient. The TAP sensor achieves high linearity (~0.99), exceptional resolution (<0.1°), and an expansive measurement range (±120 N·mm for torque and >50 N for normal force), offering bidirectional measurement with a single readout. Compared to conventional sensors, the TAP sensor features a simple structure, low cost, and streamlined fabrication, making it suitable for diverse applications. Compared to conventional 3D Hall elements, 2D Hall element arrays, or vision-based methods (*43*), the adoption of a 1D Hall element for direct torque measurement offers a cost-effective alternative (table S1).

The efficacy of the interaction-in-the-loop framework integrated with the TAP sensor was demonstrated through various forceful manipulation tasks, including precise object placement, beam balancing, and vegetable slicing. In the challenging task of placing a larger bottle (half-filled with water) upside down on a smaller bottle’s cap, the robot accurately fine-tuned the orientation, achieving successful placement in under 5 s. During the beam balancing task, the robot significantly outperformed human performance, achieving up to 15.2 times faster completion. Moreover, in the forceful manipulation of slicing tasks, the robot, guided by torque feedback, accurately replicated the looped rotary slicing strategy, enabling continuous slicing of tough vegetables, which is unattainable using conventional methods. These experimental results demonstrate the notable potential of the TAP sensor to enhance forceful manipulation in practical applications.

While the current system demonstrates robust performance in various tasks, more complex manipulations, such as in-hand dexterity, still pose challenges. Further improvements will focus on sensor miniaturization and multimodal perception to enable multipoint contact measurement while maintaining high sensitivity and more contact information. Exploring array configurations on flexible printed circuit boards and developing flexible magnetic films with high remanence could enhance contact localization and force direction recognition. In addition, optimizing magnet shape parameters and magnetic field distributions could improve resolution, even in pressure and shear sensing contexts. Integrating an additional Hall element centrally beneath the magnet may also enhance decoupling of pressure and torque outputs (fig. S35). Last, with our sensor feedback, adopting smoother and more stable robotic arm control methods may further enhance the robot’s motion performance to address more complex tasks. These advancements would enable more refined force control and dexterous manipulation, bringing robotic capabilities closer to human hand performance.

In summary, the TAP sensor and proposed interaction-in-the-loop framework substantially enhance robotic manipulation with real-time environmental feedback, advancing the field of tactile sensing and robotic dexterity. This work lays a foundation for further research into developing more adaptive and efficient robotic systems for forceful manipulation in diverse applications.

## MATERIALS AND METHODS

### Fabrication of the sensor

The cap structure is made by a 3D printer (Formlabs 3 SLA, Formlabs, US) using Elastic 80A resin. Then, the cap structure is cured in a ultraviolet curing oven at 45°C with a curing time of 10 min after being printed by a 3D printer immediately. Subsequent to the preparation of the top structure of the sensor, a layer of RTV (Kafuter k704) must be applied to its surface and left to cure completely at room temperature for 24 hours. The permanent magnet (N35, Shenzhen Lala magnet, China) is embedded in the suspended structure, which is fabricated by a 3D printer (Bambu Lab P1, China), applying a small amount of resin to the surface with a syringe in a curing oven for 1 min for encapsulation. The middle elastomer is made by Ecoflex 00-50 (Smooth-on Inc.) with good ductility and low modulus. It is mixed by Ecoflex A and Ecoflex B in a ratio of 1:1 and agitated for 10 min. According to the printer’s resolution, the mold exhibits a height tolerance of 0.08 mm. While height is a known factor influencing sensor performance, the resulting deviation in sensor output is negligible, amounting to below 0.1%. After that, the mixture is cast into the mold and cured for 2 hours at 70°C. The Hall element (DH49E, DiShi Hall Company, China) is embedded in the middle of the printed circuit board, and the sensor structure is bonded to the printed circuit board using polyacrylate. Last, all of the above will be centrally assembled into the sensor frame, thereby ensuring the cohesion of the top layer, middle layer, and bottom layer through the utilization of an adhesive substance. The plastic structural parts used in the experiments were prepared using a PLA-3D printer.

### Sensor characterization

The sensors were subjected to pressure tests using a tensile testing machine (PT-1176S, PRESSURE TESTING MACHINE, China) with a maximum capacity of 100 N and a minimum detectable force of 0.1 N. Torsion calibration was performed with a four-axis motion platform (RSP60, TOTEN, China) capable of incremental angular loading in 0.01° steps, in conjunction with a torque sensor (0 to 1 N·m) from Zhongnuo (ZMNT, China). For the response-time test, data were acquired with a Tektronix oscilloscope (MOD3; US) at a sampling frequency of 1 kHz. The Hall element was powered by an adjustable dc power supply (FPS-1502D, China).

### Demonstration setup

In the grasping task, a parallel jaw (RM, RM-GB-17-60; China) was mounted on a robotic arm (TRIAXIAL MECHANICAL ARM, China), and the end-effector finger was fabricated by 3D printing using PLA. Tactile signals were acquired and digitized with an Arduino-based system (12-bit resolution, 100-Hz sampling rate) to achieve analog-to-digital conversion.

## Supplementary Materials

### The PDF file includes:

Texts S1 to S5 Figs. S1 to S35 Tables S1 to S4 Legends for movies S1 to S5
