# Supplementary material for: Touching with torque enables human-level robotic dexterity
Source: Sci Adv. 2026 Mar 4;12(10):eaec3263. doi: 10.1126/sciadv.aec3263 (PMC12959395; doi:10.1126/sciadv.aec3263)
Supplement: Supplementary file 1 — Texts S1 to S5 Figs. S1 to S35 Tables S1 to S4 Legends for movies S1 to S5 [file sciadv.aec3263_sm.pdf]

Science Advances
AAAS

Supplementary Materials for

Touching with torque enables human-level robotic dexterity

Ling Wang *et al.*

Corresponding author: Yu Sun, yu.sun@xjtu.edu.cn; Laihao Yang, yanglaihao@xjtu.edu.cn;
Yajing Shen, eeyajing@ust.hk

Sci. Adv. **12**, eaec3263 (2026)
DOI: 10.1126/sciadv.aec3263

The PDF file includes:

Texts S1 to S5
Figs. S1 to S35
Tables S1 to S4
Legends for movies S1 to S5

Other Supplementary Material for this manuscript includes the following:

Movies S1 to S5

Text S1. Contact modeling.

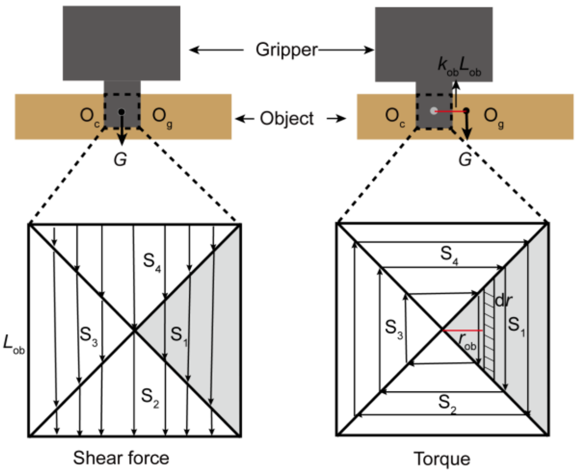

Fig. S1. Schematic diagram of the different types of forces on a contact interface.

As illustrated in Fig. S1, it is assumed that the jaws are firmly attached to the center of a uniform object. In this study, the contact surface is assumed to be a square and can be characterized by the side length L_{ob}. To simplify the calculation, the contact surface is segmented into four regions when the object is stably clamped, as depicted in Fig. S1. Two different cases are employed to illustrate the torque effect when manipulating robotics. In the first case, where the clamping point coincides with the object's gravity center (the geometrical center for homogeneous objects), uniform friction occurs on the gripper surface to withstand the tangential force *G* (Fig. S1: Left). In the second case, where the clamping point is offset from the object's gravity center by a distance $k_{ob}L_{ob}$, both shear force and torque are encountered by the gripper surface (Fig. S1: Right). Given the object's gravity center located on the right side, slipping is most probably prone to occur in the S_1 region of the contact surface. Assuming that the shear force generates an equivalent torque effect on the contact surface, the following formulations can be performed to assess the relationship between the torque, shear force, and offset distance.

It is assumed that the torque effects on four regions of the contact surface are the same. Thus, the torque on the S_1 region can be calculated as

$$T_{S_1} = \frac{k_{ob}GL_{ob}}{4} \tag{S1}$$

Assume that the equivalent tangential force in the S_1 region is P_{S_1}, then the corresponding torque is assessed as

$$T_{S_1} = \int_0^{L_{ob}/2} 2p_{S_1} r_{ob}^2 \mathrm{d}r = \frac{p_{S_1} L_{ob}^3}{12} \tag{S2}$$

Combining Eq. (S1) and Eq. (S2), we can get the equivalent shear force as Eq (S3).

$$F_{S_1} = \frac{p_{S_1} L_{ob}^2}{4} = \frac{3k_{ob}G}{4} \tag{S3}$$

where F_{S_1} denotes the equivalent tangential force. Eq. (S3) indicates that, for a given object, a larger offset distance between the clamping point and the object's gravity center may increase the tangential force significantly, thus leading to potential slippage. Therefore, it is of utmost importance to detect torsion information including the torque and torsion angle to ensure the robust and stable manipulation of objects, especially in unstructured environments.

Table. S1. Comparison of characteristics for different sensors with torsion/torque detection ability.

Sensing principle	Structure	Range	Linearity	Number of readouts	RMSE for torque /Resolution	RMSE for torsion	Response time	Anti–stray-field	Calibration burden	Cost/ Packaging complexity*
Piezoresistance	Tenon-and-mortise(*28*)	±4 N·mm	0.89	12	0.28 N·mm	NA	120 ms	NA	High	NA
	Interlock(*32*)	0~100 N·mm	0.87	1	0.2 N·mm	NA	33ms	NA	Low	NA
Capacitive	Pyramid-Plug(*14*)	0~123 N·mm	0.89	1	8.7 N·mm	NA	69 ms	NA	Low	NA
	Porous(*20*)	0~13 N·mm	NA	4	0.5 N·mm	NA	12.5 ms	NA	Medium	NA
Gelsight	DotView(*25*)	0~59 N·mm	0.92	64	3.94 N·mm	NA	30 ms	NA	High	NA
	Laminar structure(*34*)	5~150 N·mm	0.85	61	5.2 N·mm	NA	250 ms	NA	High	NA
	Spring(*24*)	± 20 N·mm	0.84	3	3.72 N·mm	NA	33 ms	NA	Medium	NA
	Hollow cylinder(*31*)	±20 N·mm	0.80	6	2.28 N·mm	NA	98 ms	NA	Medium	NA
	Laminar structure(*33*)	±40 N·mm	NA	961	8.4 N·mm	NA	NA	NA	High	NA
	Laminar structure(*35*)	±50 N·mm	NA	NA	5.6 N·mm	NA	NA	NA	NA	NA
	Laminar structure(*10*)	0~30°	NA	NA	NA	3°	NA	NA	NA	NA
	Laminar structure(*43*)	NA	NA	NA	NA	NA	NA	NA	High	>$9.50
	Laminar structure(*19*)	0~14.26°	NA	142	NA	3.96°	NA	NA	High	NA
Magnetic	Laminar structure(*36*)	±10°	0.89 (Tz)	9	NA	2°	NA	NA	Medium	>$6.0
	Laminar structure(*37*)	NA	0.96 (Tx)	8	NA	NA	NA	2 mT	Medium	>$4.0
(This work)	Interlocking sleeves	±30°/ ±120 N·mm	0.99 (Tz)	1	0.4 N·mm	0.1°	20 ms	1.14 mT	Low	<$1.0

[*] Denotes the complexity of different types of sensors is often difficult to compare in quantitative terms due to their varying packaging methods and equipment.

Text S2. Modeling and validation of magnetosensitive tactile sensor.

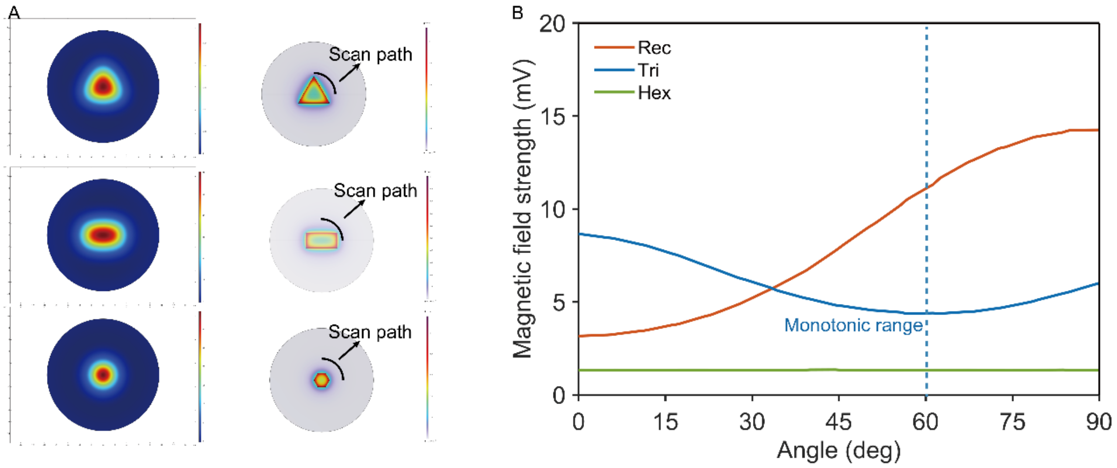

Fig. S2. Magnetic field distribution of magnets of different shapes. (A) Magnetic field distribution of three different-shaped magnets: triangle, rectangle, and hexagon. **(B)** Change in magnetic flux density along a circular path.

Hall elements can convert variations in magnetic flux density into corresponding voltage changes via the Hall effect. This voltage variation corresponds to the variation in the average magnetic flux density across the Hall element. Conventionally, the magnetic flux density at a single point in the field is often assumed to represent the average value across the element, which introduces inaccuracies in the calculated Hall voltages. Alternative approaches utilize finite element analysis with highly refined meshes to achieve high precision, albeit at the cost of significantly increased computational load. To address these challenges, we derive an expression for the average magnetic flux density variations across Hall elements and the resulting output voltage by combining partial integration with numerical integration methods, achieving both higher precision and faster computation. The detailed process is presented as follows.

A) Distribution of the magnetic field of the rectangular magnet

Based on Ampere's molecular circulation hypothesis (Fig. S3A), the magnetic field excited by a magnet once magnetized can be regarded as that of a large circular current (Fig. S3B). According to the Biot-Savart law, the magnetic flux density generated by the current element $\mathrm{d}\boldsymbol{l}$ in the current loop of a thin wire at a point (x, y, z) in space is defined as

$$\mathrm{d}\boldsymbol{B} = \frac{\mu_0}{4\pi}\frac{I\mathrm{d}\boldsymbol{l}\times\boldsymbol{r}}{r^3} \tag{S4}$$

where μ_0 is the magnetic permeability in air, r is the distance from the current element to the point P, $\boldsymbol{r}$ is the vector diameter of the current element to the point P, and I is the equivalent current strength. Setting the included angle between the direction of the current element and the direction pointing to point P as θ_{mag}, then Eq. S4 can be further simplified as

$$\mathrm{d}B = \frac{\mu_0 I\mathrm{d}l\cdot\sin\theta_{\mathrm{mag}}}{4\pi r^2} \tag{S5}$$

Let the magnetizing current density at the inner surface of the loop be J, and then the current strength of the thin layer current ring $A'B'C'D'A'$ of thickness $\mathrm{d}z_0$ is $J\mathrm{d}z_0$. In this case, the Hall element is installed parallel to the rectangular magnet ($a\times b\times h$) magnetized along the z-axis. Thus, only the component of the magnetic flux density along the z-axis (B_z) needs to be calculated. To calculate the magnetic flux density generated by the current loop $A'B'C'D'A'$ at the point

P , we divide the current loop into four current segments as $A'B'$, $B'C'$, $C'D'$, and $D'A'$, as shown Fig. S1C. Taking the one generated by the section $A'B'$ at the point P for example, it can be written as

$$\mathrm{d}B_{z_1} = \frac{\mu_0 \mathrm{J}\mathrm{d}z_0}{4\pi}\int_0^b \frac{\sin\theta_{\mathrm{mag}}\cdot\cos\varphi}{r^2}\mathrm{d}y_0 \tag{S6}$$

where φ is the angle between the β_{plane} plane and $x'o'y'$ plane.

$$\sin\theta_{\mathrm{mag}} = \frac{r_0}{r} = \frac{\sqrt{(a-x)^2+(z-z_0)^2}}{\sqrt{(x-a)^2+(y-y_0)^2+(z-z_0)^2}} \tag{S7}$$

where r_0 is the length of the projection of the vector on $\boldsymbol{r}$ the $x'o'z'$ plane.

$$\cos\varphi = \frac{a-x}{\sqrt{(x-a)^2+(z-z_0)^2}} \tag{S8}$$

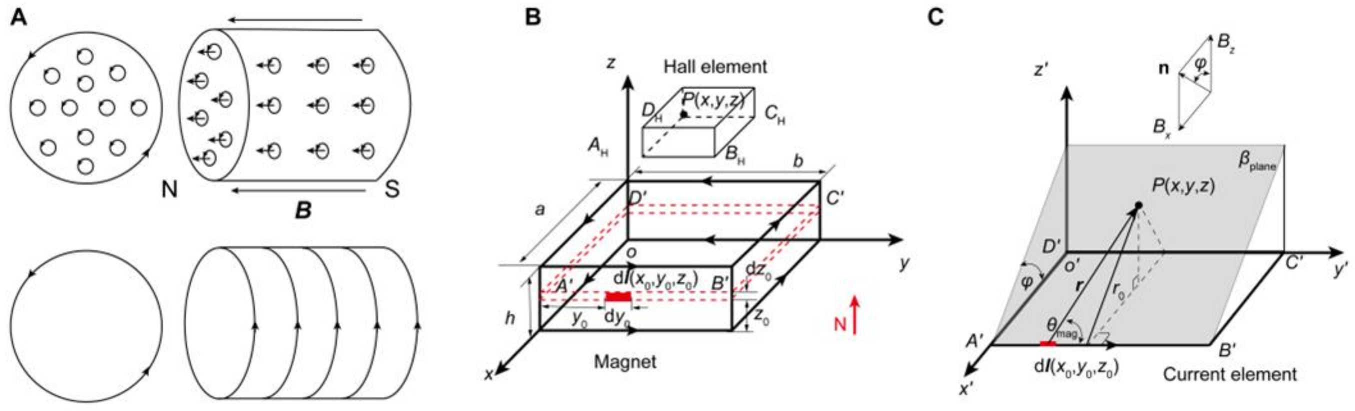

Fig. S3. Schematic diagram for solving the magnetic field distribution. (**A**) Ampere's molecular current hypothesis. (**B**) Rectangular magnet model using molecular current. (**C**) Magnetic flux density generated by the current circle.

Substituting the Eq. (S7) and Eq. (S8) into Eq. (S6), we can get

$$\mathrm{d}B_{z_1} = \frac{\mu_0 \mathrm{J}\mathrm{d}z_0}{4\pi}\int_0^b \frac{(a-x)dy_0}{[(x-a)^2+(y-y_0)^2+(z-z_0)]^{3/2}} \tag{S9}$$

Similarly, we can get the magnetic flux density $\mathrm{d}B_{z_2}$, $\mathrm{d}B_{z_3}$, $\mathrm{d}B_{z_4}$ generated by $B'C'$, $C'D'$, and $D'A'$, respectively.

$$\mathrm{d}B_{z_2} = \frac{\mu_0 \mathrm{J}\mathrm{d}z_0}{4\pi}\int_0^a \frac{(b-y)dx_0}{[(x-a)^2+(y-y_0)^2+(z-z_0)]^{3/2}} \tag{S10}$$

$$\mathrm{d}B_{z_3} = \frac{\mu_0 \mathrm{J}\mathrm{d}z_0}{4\pi}\int_0^b \frac{x dy_0}{[x^2+(y-y_0)^2+(z-z_0)]^{3/2}} \tag{S11}$$

$$\mathrm{d}B_{z_4} = \frac{\mu_0 \mathrm{J}\mathrm{d}z_0}{4\pi}\int_0^a \frac{y dx_0}{[(x-a)^2+y^2+(z-z_0)]^{3/2}} \tag{S12}$$

According to the above analysis, the component of the magnetic flux density along the z-axis from z_0 to $z_0 + \mathrm{d}z_0$ in the thin layer section at point P is derived as Eq. (S13).

$$\mathrm{d}B_z = \mathrm{d}B_{z_1} + \mathrm{d}B_{z_2} + \mathrm{d}B_{z_3} + \mathrm{d}B_{z_4} \tag{S13}$$

Assume that

$$\psi(\gamma_1,\gamma_2,\gamma_3) = \arctan\left[\frac{\gamma_1(\gamma_3-h)}{\gamma_2\sqrt{\gamma_1^2+\gamma_2^2+(\gamma_3-h)^2}}\right] - \arctan\left[\frac{\gamma_1\gamma_3}{\gamma_2\sqrt{\gamma_1^2+\gamma_3^2+\gamma_3^2}}\right] \tag{S14}$$

Thus, the expression of the magnetic flux density of the rectangular magnet at point P is

$$B_z = \int_0^h \mathrm{d}B_z = -\frac{\mu_0 J}{4\pi}[\psi(b-y,a-x,z)+\psi(a-x,b-y,z)+\psi(y,a-x,z)+\psi(a-x,y,z) \\ +\psi(b-y,x,z)+\psi(x,b-y,z)+\psi(y,x,z)+\psi(x,y,z)] \quad \text{(S15)}$$

B) Analytical expression of Hall voltage under the rectangular magnet

Here, the variation of Hall voltage is analyzed for the case of constant voltage source input. The dimension of the Hall sensor is $W \times L \times H$. The expression of the relationship between Hall voltage U_H and external constant voltage U_S can be written as

$$U_{\mathrm{H}} = \mu_{\mathrm{n}} U_{\mathrm{S}} B_{\mathrm{Hall}} \frac{W}{L} \quad \text{(S16)}$$

where μ_{n} is the carrier mobility and B_{Hall} denotes the average magnetic flux density perpendicular to the semiconductor surface. B_{Hall} can be calculated as

$$B_{\mathrm{Hall}} = \frac{1}{WL}\iint_D B_z \mathrm{d}x\mathrm{d}y \quad \text{(S17)}$$

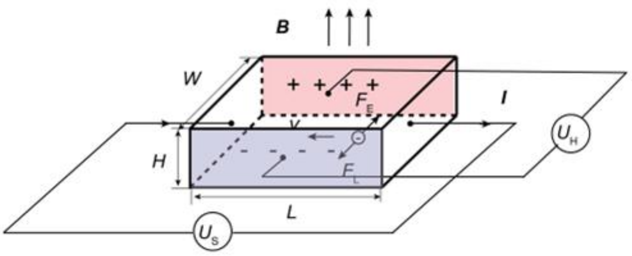

Fig. S4. Schematic diagram of the Hall effect

Assume that the coordinates of the point D_{H} is (x, y, z), as shown in Fig. S3. According to Eq. (S15), one of the components $-\frac{\mu_0 J}{4\pi}(\psi(b-y,a-x,z))$ is selected for integral operation first, where the integration area is D_0 , as shown in Fig. S5. We define that $K_0 = -\frac{\mu_0 J}{4\pi}$. Thus, the integration of this component in the region corresponding to the Hall element is B_1 and can be expressed as

$$B_1 = K_0 \iint_{D_0} \arctan[\frac{b-y}{a-x}\frac{z-h}{\sqrt{(b-y)^2+(a-x)^2+(z-h)^2}}] - \arctan[\frac{b-y}{a-x}\frac{z}{\sqrt{(b-y)^2+(a-x)^2+z^2}}]\mathrm{d}x\mathrm{d}y \quad \text{(S18)}$$

Similarly, the expressions corresponding to the remaining seven components have the same form as the first one. In order to simplify Eq. (S18), firstly, we assume that

$$B_{11} = K_0 \iint_{D_0} \arctan[\frac{b-y}{a-x}\frac{z-h}{\sqrt{(b-y)^2+(a-x)^2+(z-h)^2}}]\ \mathrm{d}x\mathrm{d}y \quad \text{(S19)}$$

$$B_{12} = -K_0 \iint_{D_0} \arctan[\frac{b-y}{a-x}\frac{z}{\sqrt{(b-y)^2+(a-x)^2+z^2}}]\ \mathrm{d}x\mathrm{d}y \quad \text{(S20)}$$

Then, the following coordinate transformation is performed to simplify the above expressions. To obtain the simplified expression of B_{11}, it is assumed that $Y = \frac{b-y}{z-h}$ and $X = \frac{a-x}{z-h}$ to construct a new coordinate system $X''O''Y''$, as

shown in Fig. S5. Thus, D_0 is transformed into a new integration area. Correspondingly, we have $X_{11}=\frac{a-x}{z-h}$, $X_{12}=\frac{a-x-W}{z-h}, Y_{11}=\frac{b-y}{z-h}, Y_{12}=\frac{b-y-L}{z-h}$, respectively. Then, we can derive the following simplified expression of B_{11} as

$$B_{11}=K_0(z-h)^2\iint_{D_{11}}\arctan[\frac{Y}{X}\frac{1}{\sqrt{Y^2+X^2+1}}]\,\mathrm{d}X\mathrm{d}Y \tag{S21}$$

Similarly, B_{12} is simplified as

$$B_{12}=-K_0z^2\iint_{D_{12}}\arctan[\frac{Y}{X}\frac{1}{\sqrt{Y^2+X^2+1}}]\,\mathrm{d}X\mathrm{d}Y \tag{S22}$$

Hence, B_1 can be written as:

$$B_1=B_{11}+B_{12}=K_0(z-h)^2\iint_{D_{11}}\arctan[\frac{Y}{X}\frac{1}{\sqrt{Y^2+X^2+1}}]\,\mathrm{d}X\mathrm{d}Y-K_0z^2\iint_{D_{12}}\arctan[\frac{Y}{X}\frac{1}{\sqrt{Y^2+X^2+1}}]\,\mathrm{d}X\mathrm{d}Y \tag{S23}$$

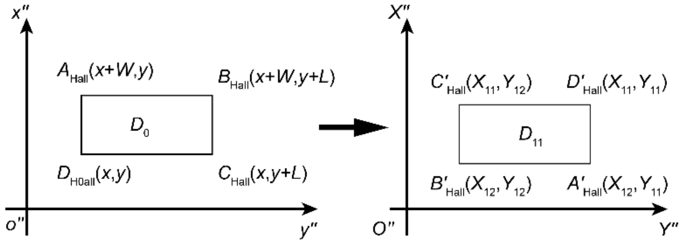

Fig. S5. Coordinate transformation.

In the same way, we can derive a simplified expression of the second term of Eq. (S15), which can be written as

$$B_2=B_{21}+B_{22}=K_0(z-h)^2\iint_{D_{21}}\arctan[\frac{X}{Y}\frac{1}{\sqrt{Y^2+X^2+1}}]\,\mathrm{d}X\mathrm{d}Y-K_0z^2\iint_{D_{22}}\arctan[\frac{X}{Y}\frac{1}{\sqrt{Y^2+X^2+1}}]\,\mathrm{d}X\mathrm{d}Y \tag{S24}$$

where, the integration interval D_{21} is the same as D_{11} and D_{22} is the same as D_{12}.

The remaining components of Eq. (S15) can be simplified using the same approach. Moreover, we found that B_1, B_3, B_5 and B_7 have the same simplified expressions as Eq. (S23), while B_2, B_4, B_6 and B_8 can be simplified as Eq. (S24). The integral interval of each term is as follows:

Table. S2. Integral intervals for each term.

D_{11}, D_{21}	$X_{11}=\frac{a-x}{z-h}, X_{12}=\frac{a-x-W}{z-h}, Y_{11}=\frac{b-y}{z-h}, Y_{12}=\frac{b-y-L}{z-h}$
D_{12}, D_{22}	$X_{21}=\frac{a-x}{z}, X_{22}=\frac{a-x-W}{z}, Y_{21}=\frac{b-y}{z}, Y_{22}=\frac{b-y-L}{z}$
D_{31}, D_{41}	$X_{31}=\frac{a-x}{z-h}, X_{32}=\frac{a-x-W}{z-h}, Y_{31}=\frac{y+L}{z-h}, Y_{32}=\frac{y}{z-h}$
D_{32}, D_{42}	$X_{41}=\frac{a-x}{z}, X_{42}=\frac{a-x-W}{z}, Y_{41}=\frac{y+L}{z}, Y_{42}=\frac{y}{z}$
D_{51}, D_{61}	$X_{51}=\frac{x}{z-h}, X_{52}=\frac{x+W}{z-h}, Y_{51}=\frac{b-y-L}{z-h}, Y_{52}=\frac{b-y}{z-h}$

D_{52}, D_{62}	$X_{61}=\frac{x}{z}, X_{62}=\frac{x+W}{z}, Y_{61}=\frac{b-y-L}{z}, Y_{62}=\frac{b-y}{z}$
D_{71}, D_{81}	$X_{71}=\frac{x}{z-h}, X_{72}=\frac{x+W}{z-h}, Y_{71}=\frac{y}{z-h}, Y_{72}=\frac{y+L}{z-h}$
D_{72}, D_{82}	$X_{81}=\frac{x}{z}, X_{82}=\frac{x+W}{z}, Y_{81}=\frac{y}{z}, Y_{82}=\frac{y+L}{z}$

We arrange the above formula according to the same integral interval and define the following expression.

$$M_D(X,Y)=\iint_D \arctan[\frac{X}{Y}\frac{1}{\sqrt{Y^2+X^2+1}}]\,\mathrm{d}X\mathrm{d}Y+\iint_D \arctan[\frac{Y}{X}\frac{1}{\sqrt{Y^2+X^2+1}}]\,\mathrm{d}X\mathrm{d}Y \tag{S25}$$

For a rectangular magnet, the average magnetic flux density passing through the Hall element can be written as

$$\begin{aligned}B_{\mathrm{Hall}}=\frac{K_0}{WL}[&(z-h)^2\cdot(M_{D_{11}}(X,Y)+M_{D_{31}}(X,Y)+M_{D_{51}}(X,Y)+M_{D_{71}}(X,Y))\\&-z^2(M_{D_{12}}(X,Y)+M_{D_{32}}(X,Y)+M_{D_{52}}(X,Y)+M_{D_{72}}(X,Y))]\end{aligned} \tag{S26}$$

C) Partial integration for magnetic flux density

Since all the components of B_{Hall} share the same expression showcased by $M_D(X,Y)$, the average magnetic flux density indicated by B_{Hall} can be simplified to derive the double integral indicated by $M_D(X,Y)$. Considering the symmetry form of these two terms for $M_D(X,Y)$, we may obtain the basic form of the first-step integral as

$$\int \arctan\frac{x}{y\sqrt{x^2+y^2+1}}\mathrm{d}x = x\arctan\frac{x}{y\sqrt{x^2+y^2+1}}+y\ \mathrm{artanh}\sqrt{x^2+y^2+1}+c \tag{S27}$$

Then, we derive the analytical expressions of the first-step integral for these two terms in $M_D(X,Y)$, respectively.

$$f_1(X,Y)=X\arctan\frac{X}{Y\sqrt{X^2+Y^2+1}}+Y\ \mathrm{artanh}\sqrt{X^2+Y^2+1}+c_1 \tag{S28}$$

$$f_2(X,Y)=Y\arctan\frac{Y}{X\sqrt{X^2+Y^2+1}}+X\ \mathrm{artanh}\sqrt{X^2+Y^2+1}+c_2 \tag{S29}$$

As a result, the double integral of $M_D(X,Y)$ is decoupled after the first-step analytical integration operation and can be expressed as

$$M_D(X,Y)=N_D(Y)+N_D(X)=\int_{Y_1}^{Y_2}[f_1(X_2,Y)-f_1(X_1,Y)]\mathrm{d}Y+\int_{X_1}^{X_2}[f_2(X,Y_2)-f_2(X,Y_1)]\mathrm{d}X \tag{S30}$$

where $N_D(Y)$ and $N_D(X)$ are intermediate variables, corresponding to the following expression, respectively.

$$\begin{cases}N_D(Y)=\int_{Y_1}^{Y_2}[f_1(X_2,Y)-f_1(X_1,Y)]\mathrm{d}Y\\N_D(X)=\int_{X_1}^{X_2}[f_2(X,Y_2)-f_2(X,Y_1)]\mathrm{d}X\end{cases} \tag{S31}$$

It is hard to further integrate $N_D(Y)$ and $N_D(X)$ analytically due to the hyperbolic tangent function. Therefore, the Lomborg numerical integration algorithm is employed to derive the numerical solution of $M_D(X,Y)$. Taking $N_D(Y)$ for example, we can derive the Lomborg integration algorithm as follows.

$$T_0(0)=\frac{Y_2-Y_1}{2}[N_D(Y_1)+N_D(Y_2)] \tag{S32}$$

$$T_0(k)=\frac{1}{2}T_0(k-1)+\frac{Y_2-Y_1}{2^k}\sum_{j=0}^{2^{k-1}-1}N_D(Y_1+(2j+1)\frac{Y_2-Y_1}{2^k}) \tag{S33}$$

where k denotes the iteration number. The extrapolation acceleration formula is:

$$T_m(k-1)=\frac{1}{4^m-1}[4^m T_{m-1}(k)-T_{m-1}(k-1)],\ m=1,\ 2,\ 3;\ k=1,\ 2,\ 3,\dots \tag{S34}$$

where m is the sequence of extrapolation formulas, k is the number of iterations which can be determined by $|T_3(k)-T_3(k-1)|<0.0001$. $T_{3Y}(k)$ is the final integral result of $N_D(Y)$. Ignoring the numerical error, we have

$$N_D(Y)=T_{3Y}(k) \tag{S35}$$

As the same as Eq. (S35), we can get

$$N_D(X)=T_{3X}(k) \tag{S36}$$

Combining Eq. (S30), (S35), and (S36), we can deduce that:

$$M_D(X,Y)=N_D(Y)+N_D(X)=T_{3Y}(k)+T_{3X}(k)=T_D(X,Y) \tag{S37}$$

Therefore, at any position in space, the average magnetic flux density across the Hall element can be then calculated as:

$$\begin{aligned}B_{\text{Hall}}(x,y,z)=\frac{K_0}{WL}[(z-h)^2\cdot(T_{D_{11}}(X,Y)+T_{D_{31}}(X,Y)+T_{D_{51}}(X,Y)+T_{D_{71}}(X,Y))\\-z^2(T_{D_{12}}(X,Y)+T_{D_{32}}(X,Y)+T_{D_{52}}(X,Y)+T_{D_{72}}(X,Y))]\end{aligned} \tag{S38}$$

Substituting Eq. (S38) into Eq. (S16), we can finally get the voltage generated by the linear Hall element driven by constant voltage in the magnetic field excited by the rectangular magnet as follows:

$$\begin{aligned}U_{\text{H}}=\frac{K_0\mu_n U_S}{L^2}[(z-h)^2\cdot(T_{D_{11}}(X,Y)+T_{D_{31}}(X,Y)+T_{D_{51}}(X,Y)+T_{D_{71}}(X,Y))\\-z^2(T_{D_{12}}(X,Y)+T_{D_{32}}(X,Y)+T_{D_{52}}(X,Y)+T_{D_{72}}(X,Y))]\end{aligned} \tag{S39}$$

D) The theoretical model of the sensor

To better describe the relationship between the torsion angle and the Hall voltage, it is represented here in the cylindrical coordinate system, as shown in Fig. 2A. Accordingly, we have the following expressions between the transformation between the Cartesian coordinate system (*o-xyz*) and cylindrical coordinate system (*o-rz*)

$$\begin{cases}x=R\cos\theta_{\text{B}}\\ \text{y}=R\sin\theta_{\text{B}}\end{cases} \tag{S40}$$

where R denotes the distance between the center of the magnet and the center of the Hall element at top view. θ_{B} is the angle between the line from the center of the magnet to the Hall element and the long axis of the magnet at the top view. Therefore, the relationship between Hall voltage and torsion angle can be expressed as follows.

$$U_{\text{H}}=K_{\text{H}}B_{\text{Hall}}(x,y,z)=K_{\text{H}}B_{\text{Hall}}(R\cos\theta_{\text{B}},R\sin\theta_{\text{B}},z) \tag{S41}$$

where $K_{\text{H}}=\frac{\mu_n U_s W}{L}$ is the coefficient of the Hall element, which correlates only with the material's electrical properties.

For a certain TAP sensor, the initial angle α is constant, thus the relationship between the output voltage of the TAP sensor and the revolution angle θ can be expressed as

$$U_{\text{H}}=K_{\text{H}}B_{\text{Hall}}\ (R\cos\theta_{\text{B}},R\sin\theta_{\text{B}},z)|_{\theta_{\text{B}}=\alpha}^{\theta_{\text{B}}=\alpha+\theta} \tag{S42}$$

E) Validation

To validate the above theoretical analysis, a Gaussmeter (TD8620) is employed to measure the magnetic flux density. In this study, the Hall element (DH49E) is adopted to construct the TAP sensor and thus employed for the calculation of the average magnetic flux density. The calibrated sensitive area of this Hall element is (W: 0.35mm, L:0.35mm). To

comprehensively compare the calculation and experiment results, three magnets with different dimensions are involved in the analysis. The details of the dimensions and meshes (divided under a CCD microscope) for these magnets are shown in Table. S3. One should note that the calculation results are obtained at the center of each region. The comparative results are showcased in Fig. S6. It is illustrated that the theoretical result matches well with experiment one (the fitting coefficient reaches about 0.95), indicating the effectiveness of the theoretical model presented in the last subsection.

Table. S3. Magnet dimensions.

Dimension / Magnet	*a* (mm)	*b* (mm)	*h* (mm)
Type-I	4	3	2
Type-II	6	4	2
Type-III	6	3	2

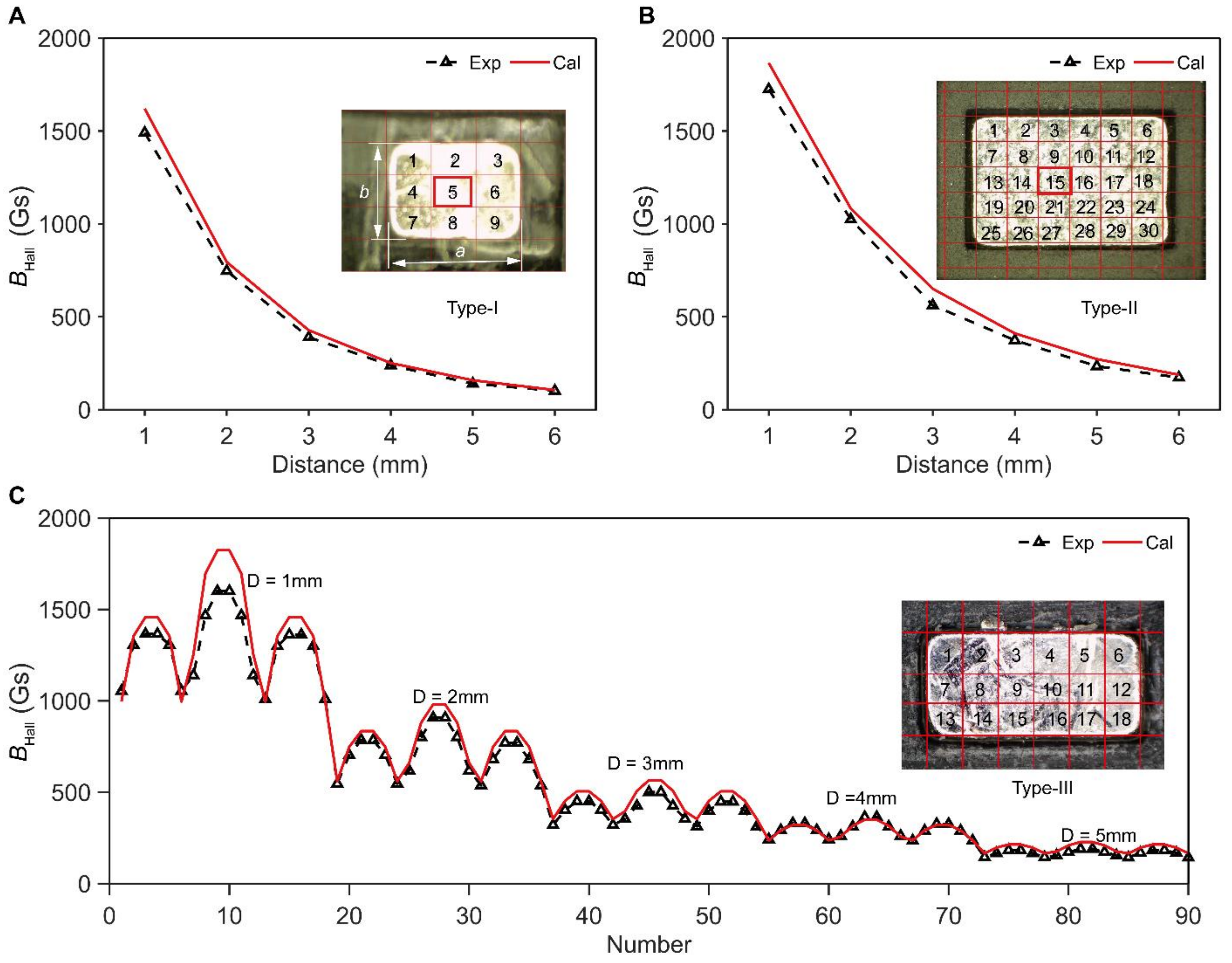

Fig. S6. Validation of the formula for the relationship between the magnet and Hall element.. (**A**) The experimental values and calculation values for magnet Type-I at point 5 for different distances between the Gaussian gauge and magnet along the *z*-axis. (**B**) The experimental values and calculation values for magnet Type-II at point 15. (**C**) The experimental values and calculation values for magnet Type-III.

Text S3. Pressure measurement mechanism.

According to the design principle of the TAP sensor and the above theoretical analysis, the TAP sensor captures pressure information through the change of the localized magnetic flux density detected by the Hall element caused by the normal pressure. Therefore, the working range of the TAP sensor for normal displacement depends on the range of the normal magnetic flux density, which is determined by the normal distance *d* between the magnet and the Hall element. To balance sensitivity while avoiding saturation, the Hall element must operate within an optimal magnetic flux density region: above the Hall limit (B_{limit} = 0.58 Gs) and below the magnetic flux density saturation threshold (1000 Gs). To achieve this, we optimized the normal distance *d*. To prevent saturation, *d* must be greater than 2 mm when the magnet and Hall element are centrally aligned. To avoid a decrease in sensitivity resulting from an increase in the normal distance, the initial normal distance *d* is set to 5 mm, where the gradient of normal magnetic flux density reduces to 17.2% relative to the gradient at 2 mm (Fig. S8). Therefore, the maximum range of normal displacement, Δd, is 3 mm. Based on this, the structural parameters of the annular elastomer (Ecoflex 00-50) between the magnet and the Hall element of the sensor can be determined as: inner diameter of 15 mm, outer diameter of 23 mm, and height of 8 mm. Specifically, the maximum range of normal displacement Δd is 3mm. Correspondingly, the normal force measurement range is 57 N (Fig. S9). Within the range of 0~15 N, the sensitivity of the normal force reaches -11.17 mV/N (21.05 kPa^{-1}).

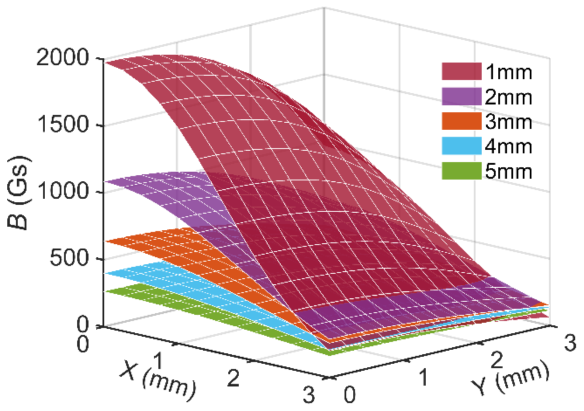

Fig. S7. Schematic of the magnetic field distribution of the rectangular magnet in the *XOY* plane under different distances.

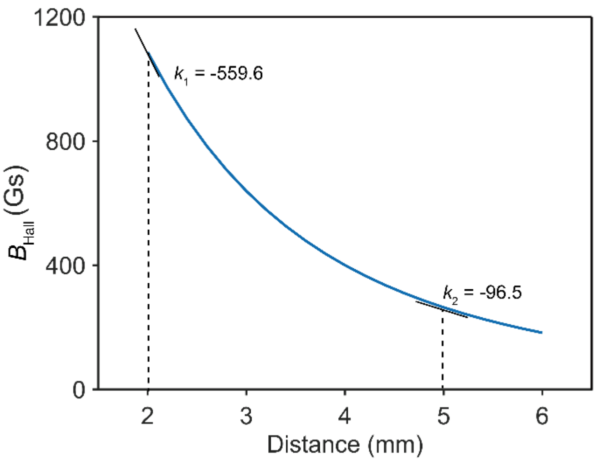

Fig. S8. Schematic of the magnetic flux density along the *z*-axis.

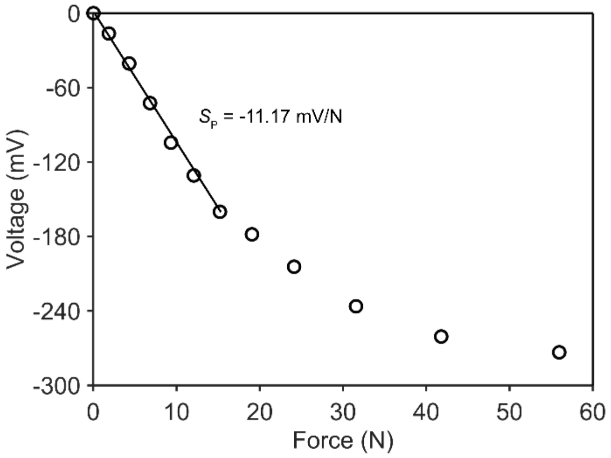

Fig. S9. The relationship between the output voltages and the applied normal force.

Text S4. Sensor design optimization and performance demonstration.

The spatial offset parameters (R, d, α), mainly affecting the variation of circumferential magnetic flux density for a certain rectangular magnet, are elaborately designed to further optimize the performance of the TAP sensor. Intrinsically, the Hall limit determines the torsion angle resolution, which is defined as the minimum torsion angle and expressed as $\Delta\theta_{\min} = \frac{B_{\text{limit}}}{\mathrm{d}B_{\text{Hall}}/\mathrm{d}\theta}$, where $\mathrm{d}B_{\text{Hall}}/\mathrm{d}\theta$ denotes the magnetic flux density gradient. It indicates that the larger $\mathrm{d}B_z/\mathrm{d}\theta$, the higher the resolution can be achieved. As illustrated in Fig. 2B and Fig. S11, the circumferential flux density gradient for the selected magnet initially increases and then decreases with the increasing R, achieving the peak value at 3.5 mm. Therefore, the radius R is set to 3.5 mm to ensure the highest design resolution of the torsion angle.

According to the working principle of the TAP sensor, pre-pressure may change the initial distance d and thus affect the dynamic resolution of the torsion angle. The variation of magnetic flux density with the changing d (from 2 mm to 5 mm) is comparatively analyzed to explore this influence mechanism. The results are showcased in Fig. S12, indicating that the best resolution is theoretically equal to 0.09°, which can be obtained when d = 2 mm. Considering the measurement noise, we set the resolution of the torsion angle as 0.1°. In this study, by setting the linearity as 0.995, the linear range can be selected as 15°~75°, as illustrated in Fig. 2C and Fig. S13. To obtain the bidirectional torsion perception function, another important capability for a soft torsion sensor is the bidirectional torsion perception. The initial angle α should be elaborately designed. In this study, we set α to be 45° by considering the symmetrical property of the circumferential magnetic flux density, where the corresponding measurement of angle θ_B is initiated as 0°. Moreover, the elastomer with a higher modulus can further broaden the measurement range.

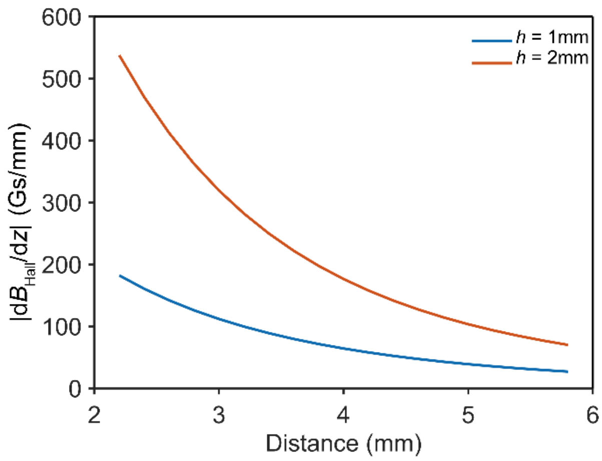

Fig. S10. Variation of the magnetic flux density gradient for different thicknesses of the magnet.

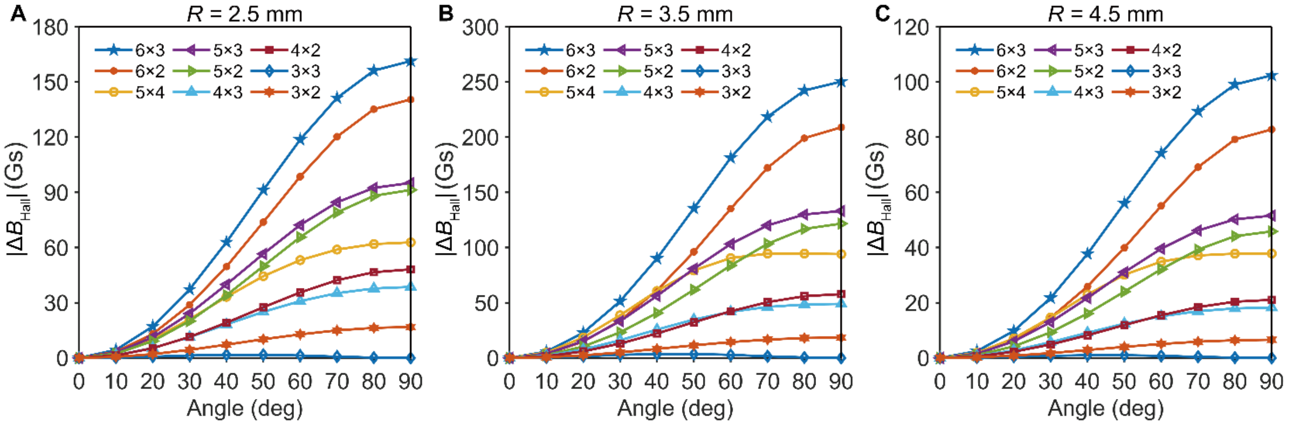

Fig. S11. Comparison of the magnetic flux density variations at different offset distances. (**A**) Radius is 2.5 mm. (**B**) Radius is 3.5 mm. (**C**) Radius is 4.5 mm.

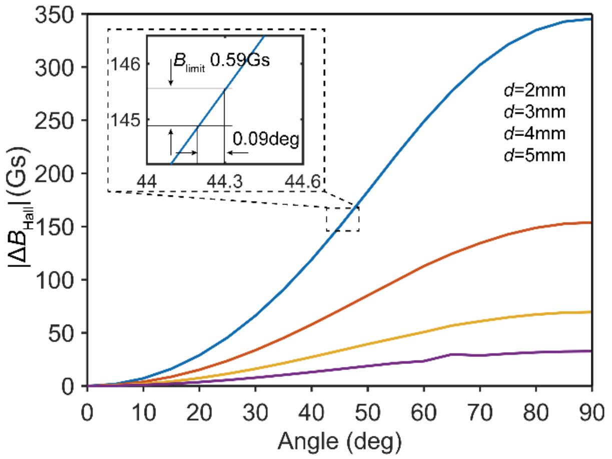

Fig. S12. Variation of the corresponding circumferential magnetic flux density at different distances.

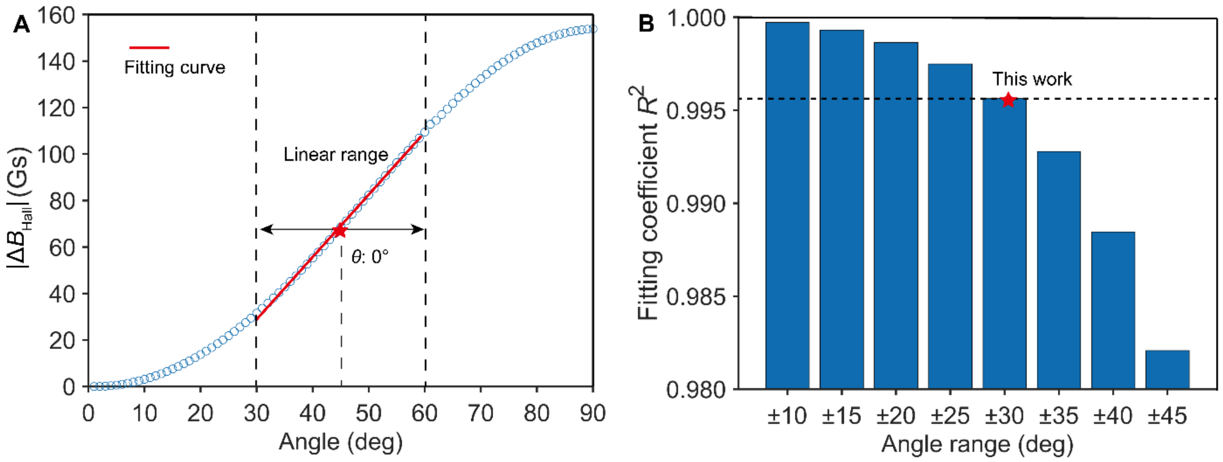

Fig. S13. Linearity analysis of the TAP sensor. (**A**) Variation of circumferential magnetic flux density with different angles θ_B. (**B**) Degree of linear fit corresponding to different working intervals.

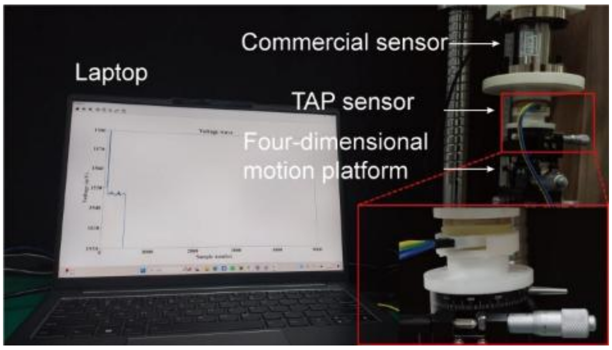

Fig. S14. Calibration platform.

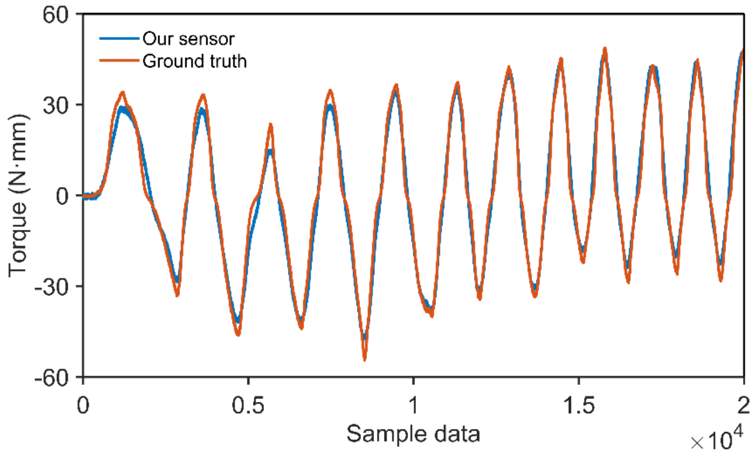

Fig. S15. Comparison of torque performance between commercial sensor and our sensor using a calibration connector.

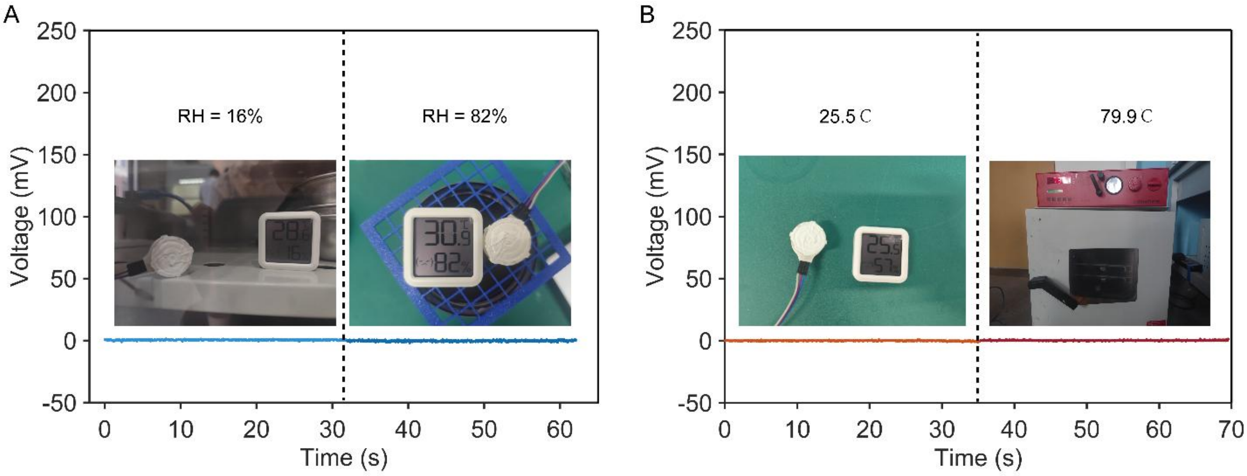

Fig. S16. The sensor output under different humidity and temperatures. (A) The output of the TAP sensor under different humidity conditions. **(B)** The output of the TAP sensor under different temperatures.

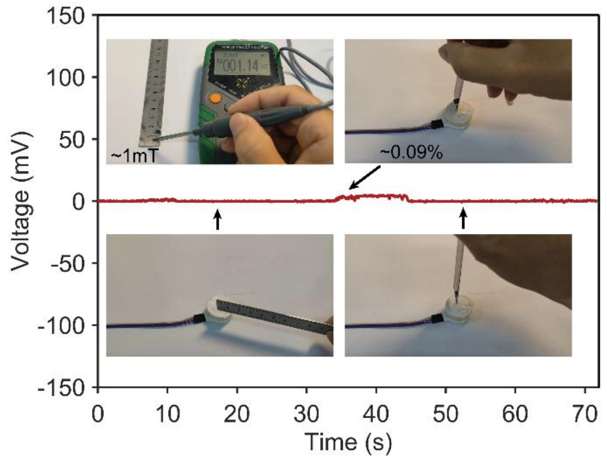

Fig. S17. Testing of the sensor in the stray magnetic fields of 1.14 mT.

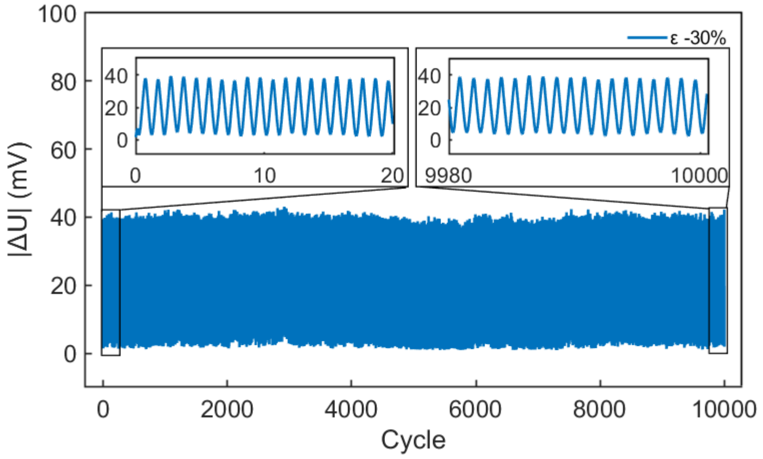

Fig. S18. 10,000 cycles of load testing.

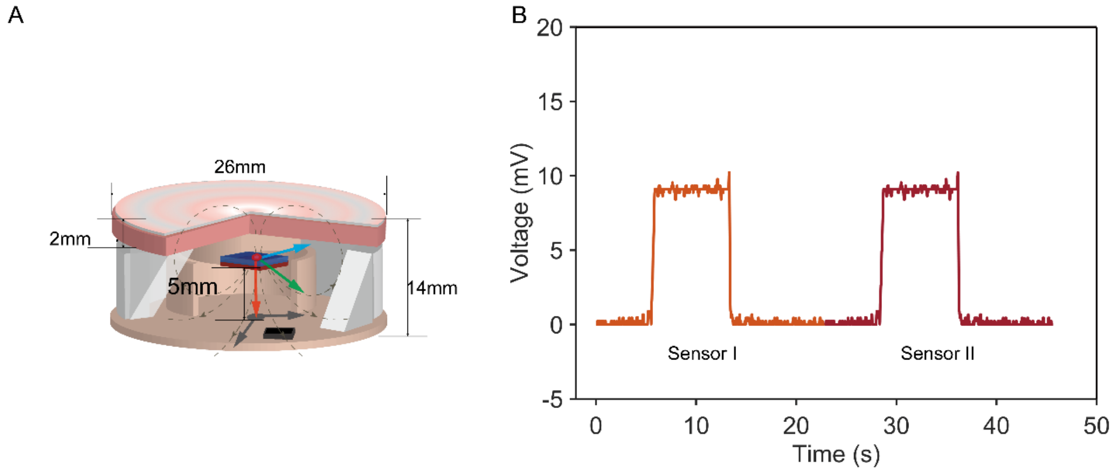

Fig. S19. Comparison of sensors from different batches. (A) Schematic diagram of the overall dimensions of the sensor. **(B)**The response of sensor-I and sensor-II under the same loading test.

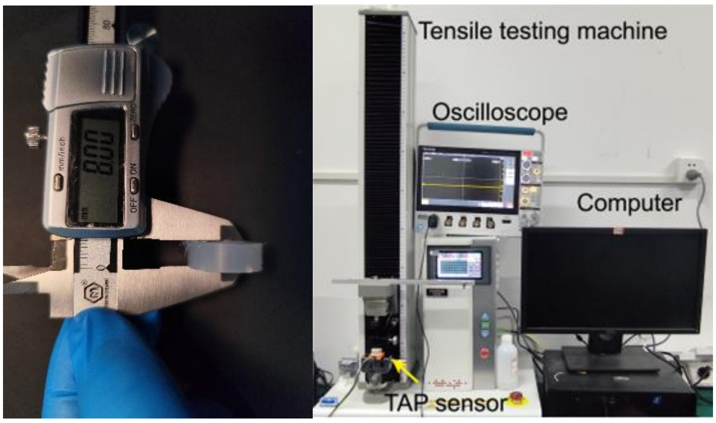

Fig. S20. The intermediate elastic layer and the response time testing platform.

Text S5. Experiment.

For comparison, 12 volunteers (9 males and 3 females, aged 22~26 years old) were invited to participate in the balance beam experiment without visual guidance. Considering the common manual operation in daily life or games, two groups of experiments are designed. In the first group, all volunteers could execute only a single grasp and make a single-direction adjustment, while the second group allowed for multiple grasps and multi-directional adjustments. The single-direction adjustment process contains the following steps: First, grasp the balance beam and move toward the board but stop once a collision between the beam and board is detected; Second, judge the orientation of the next step; Then, along with the determined direction, go on moving and simultaneously evaluating the balance state of the beam. As for the robot, this process is facilitated by the TAP sensor feedback, while, for the volunteer, it is conducted by using human-hand sensing. The multi-directional adjustments allow multiple grasps, adjustments, and movements. In the experiment, each volunteer repeated the same task of balance beam stacking 10 times in each group (Movie S4). Throughout all experiments, the total time for each attempt, starting when the beam made contact with the thin board and ending immediately upon its release, and the average success rate for every volunteer are recorded and compared. The results are illustrated in Fig. S23. Accordingly, it should be noted that the average success rate for the first group is less than 20% (Fig. S23A, B), while it increases to more than 80% for the second group (Fig. S23C, D), which is achieved by the enhancement of multiple attempts.

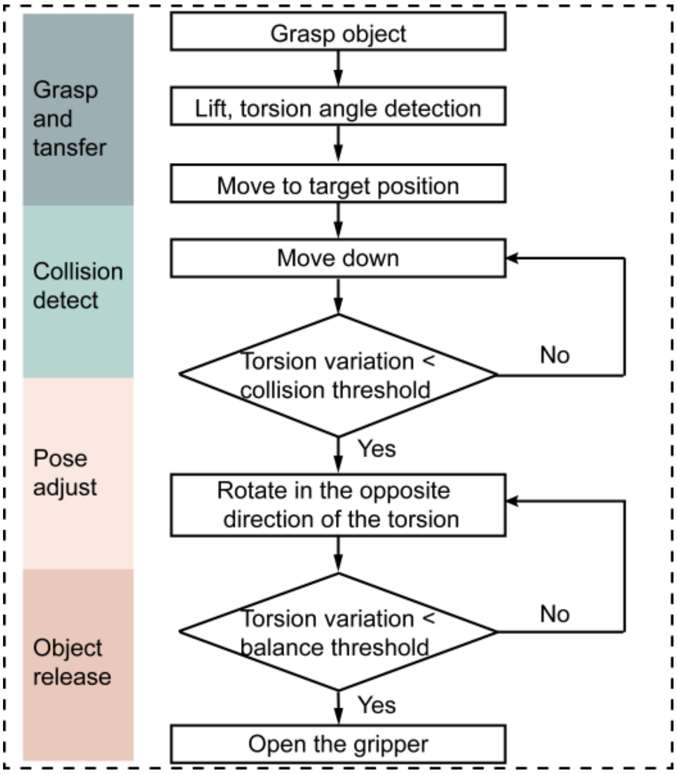

Fig. S21. Flowchart of the robot to achieve stable placement.

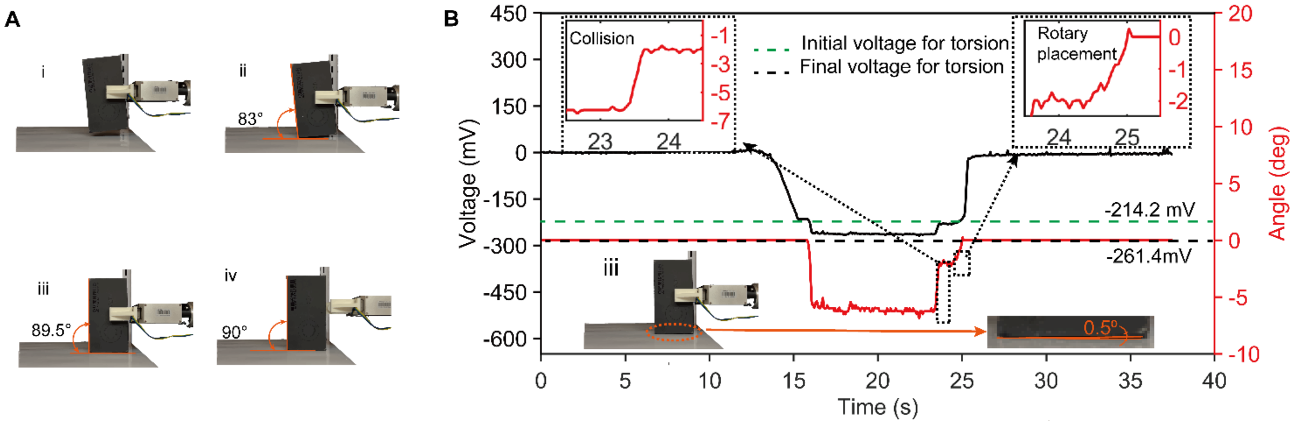

Fig. S22. Placement for black box. (**A**) The process of black box placement. (**B**) The variations of tilt angle and voltage during placement.

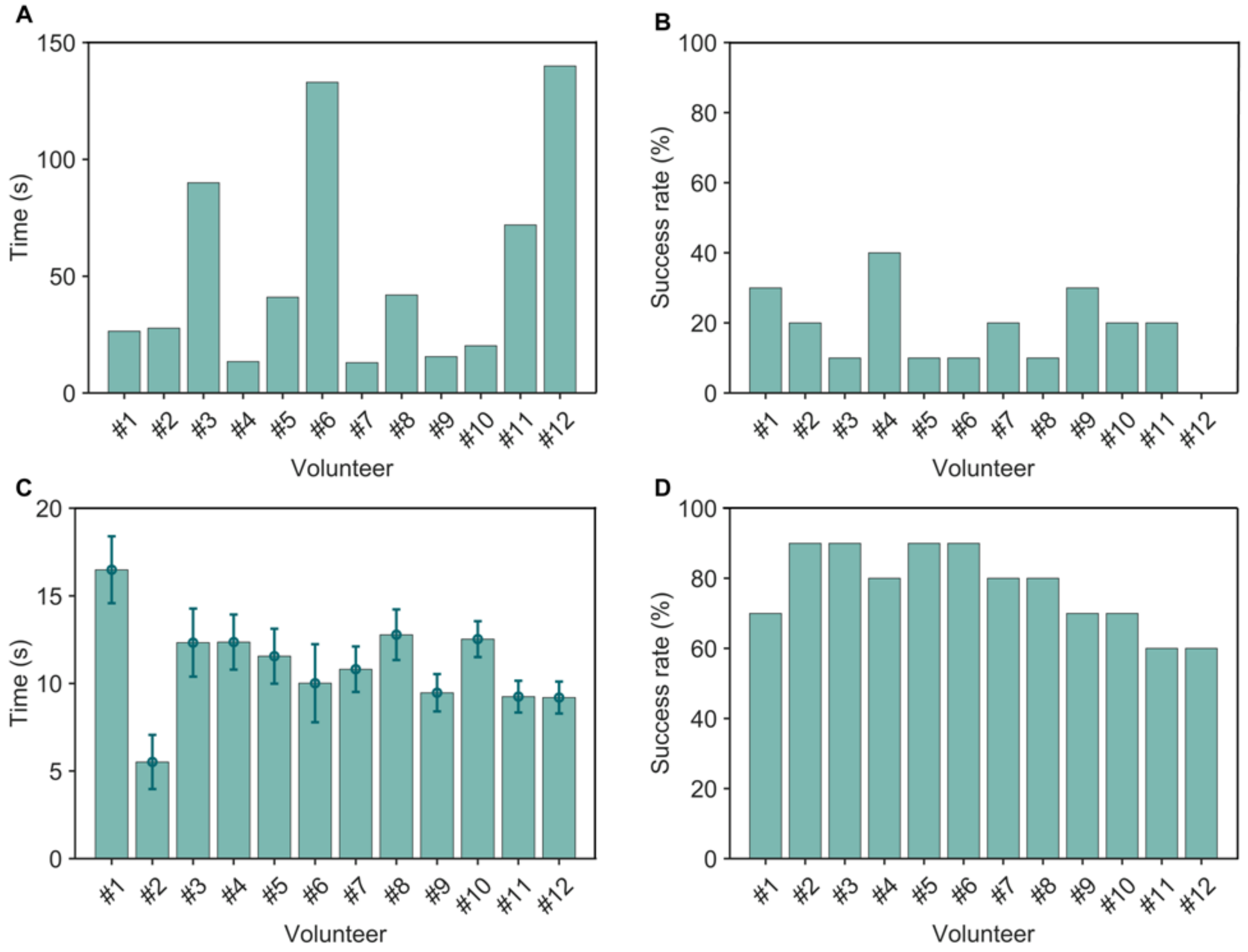

Fig. S23. Twelve volunteers place balance beam without vision. (**A**) Without allowing multiple adjustments, the time it took for each volunteer to successfully place the balance beam on the acrylic board. (**B**) Without allowing adjustments, the success rate for each volunteer in successfully placing a balance beam on the acrylic board. (**C**) Allowing multiple adjustments, the time it took each volunteer to successfully place the balance beam on the acrylic board. A total of ten tests were performed, and the error bars represent the standard error SD. (**D**) Allowing multiple adjustments, the success rate for each volunteer in this placing task.

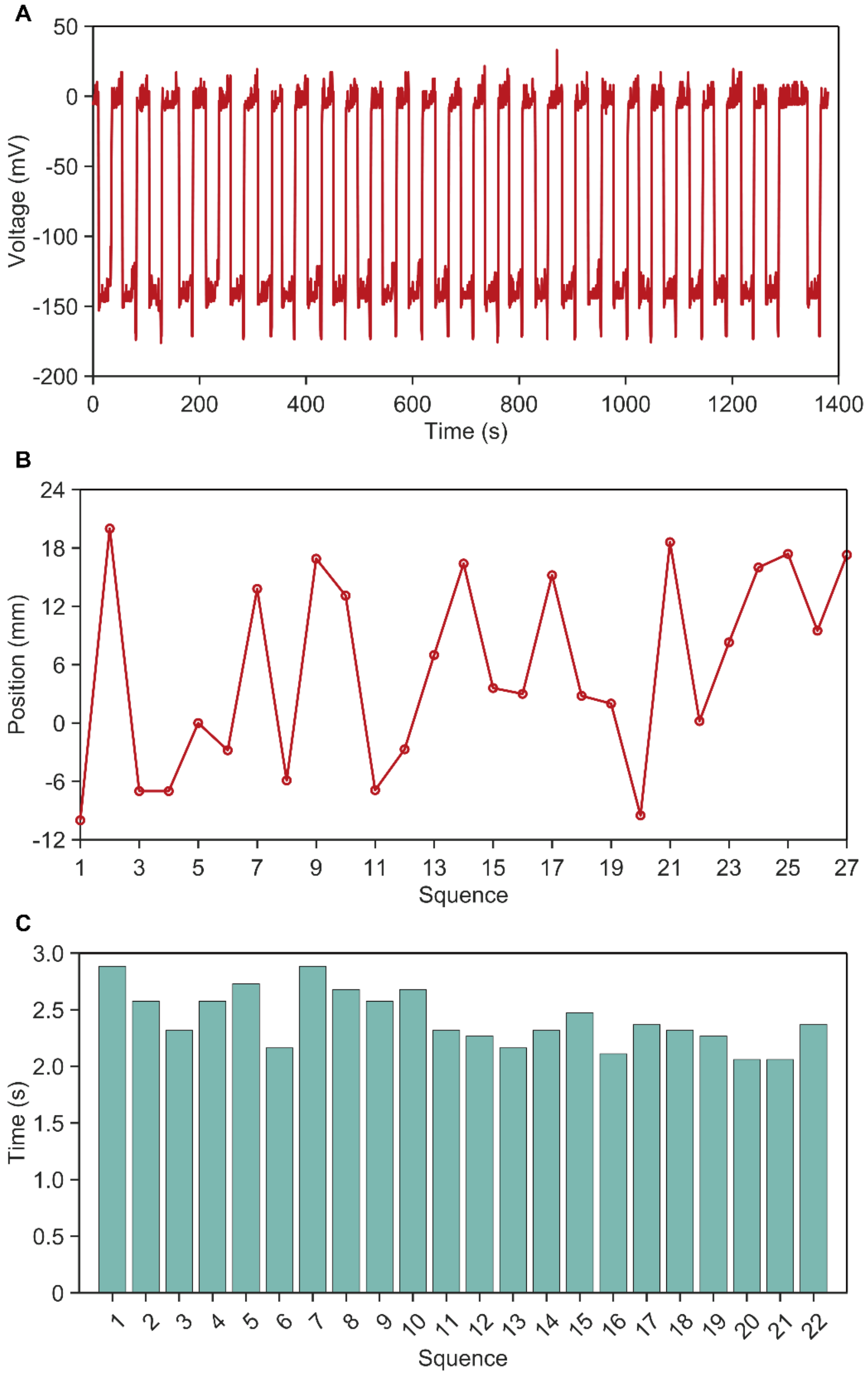

Fig. S24. Balance beam test: 27 randomized experiments. (**A**) Diagram of voltage variation in balance beam experiment for robot. (**B**) Diagram of random drop point locations for 27 experiments. (**B**) Time spent during each of the 22 successfully placed experiments.

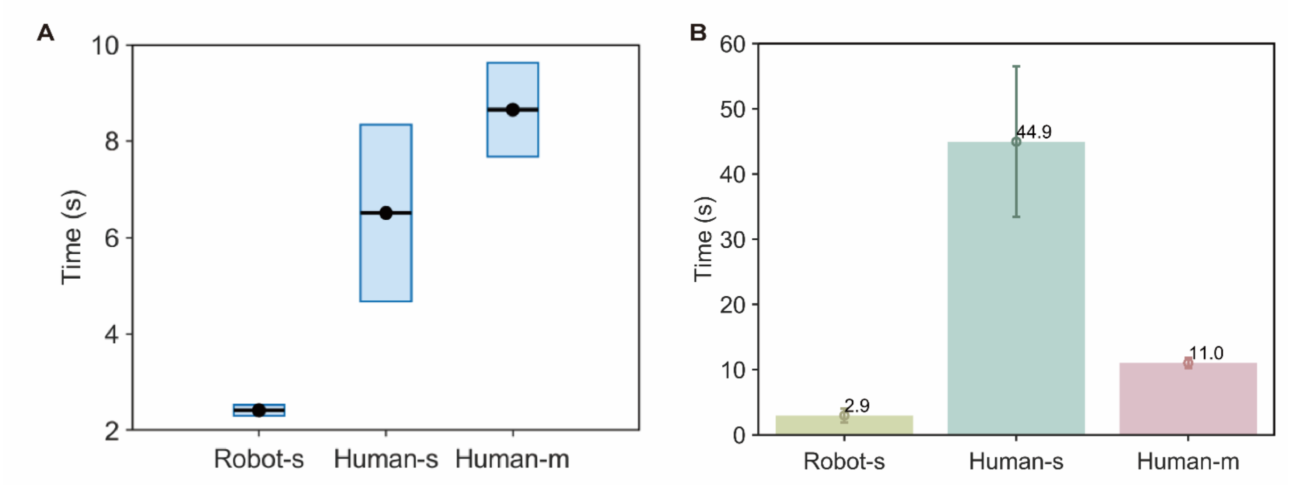

Fig. S25. Time statistics for placement task. (A) When considering solely the comparison of time required for success, the upper and lower bounds represent a 95% confidence interval. **(B)** The normalized time required to complete the task.

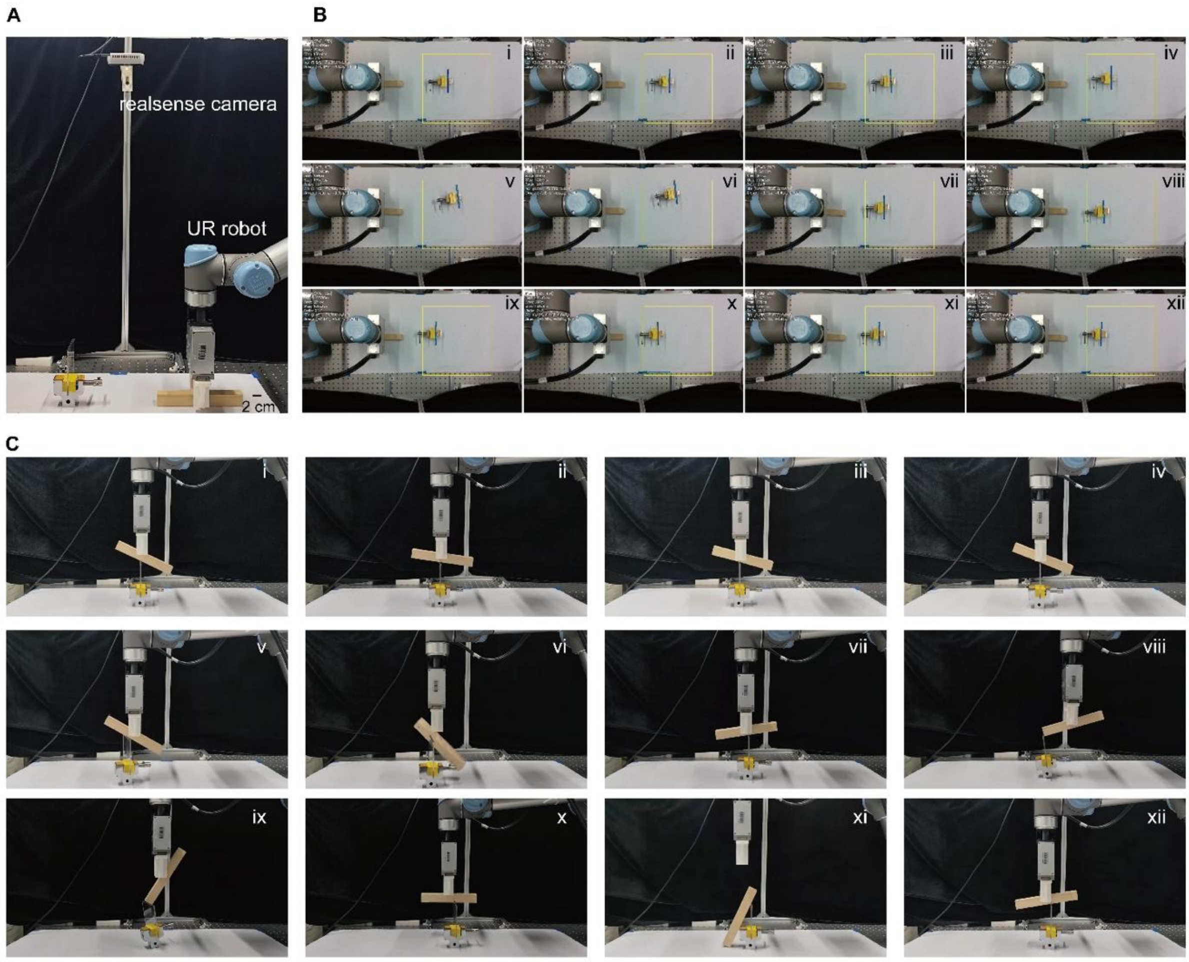

Fig. S26. Placement of the balance beam through vision guidance. (A) Schematic diagram of the vision-based placement system. **(B)** The support platform was detected by the RealSense camera with a resolution of 1280×960 during 12 placement experiments. **(C)**The result of balance beam placement during 12 placement experiments.

Table. S4. The vertical and lateral errors of the placement task based on vision. (Unit: mm)

	1	2	3	4	5	6	7	8	9	10	11	12	Average error
Vertical error	19	6	0	7	1	7	2	0	5	6	4	1	4.8
Lateral error	6.5	4.3	4.3	3.2	23.1	16.7	10.0	8.6	12.9	18.0	29.3	16.1	12.7

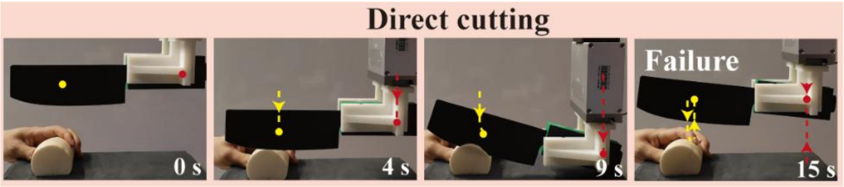

Fig. S27. Schematic diagram of slicing daikon in a direct cutting way.

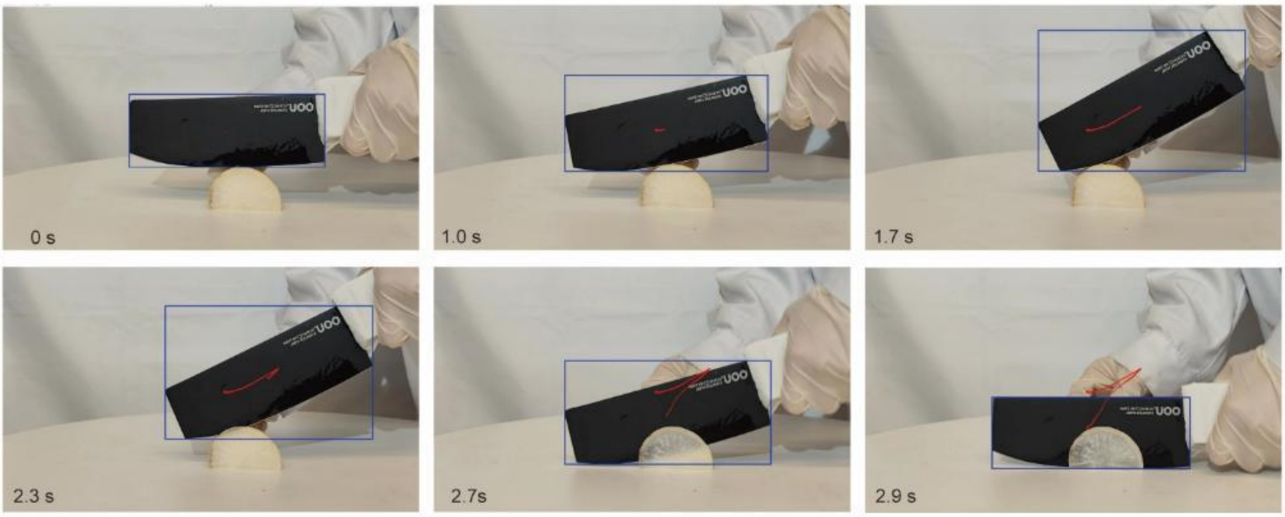

Fig. S28. Schematic diagram of the process of cutting vegetables manually.

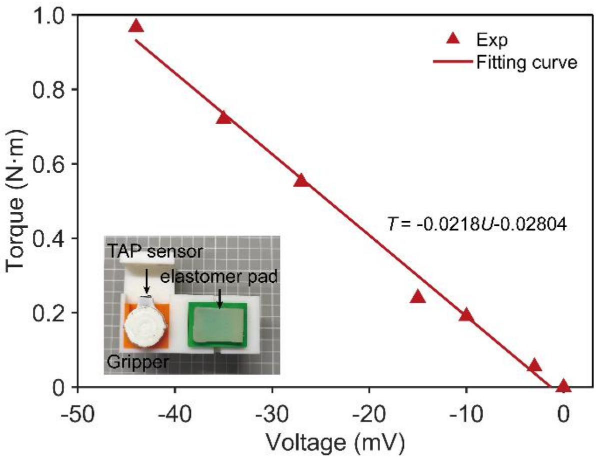

Fig. S29. Schematic diagram of the calibration of the sensor which is mounted on specific parallel jaws.

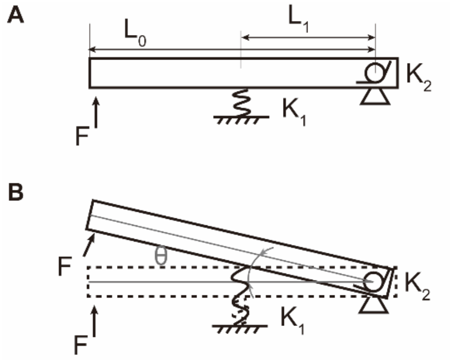

Fig. S30. The model of the end-effector. (A) The device consists of a tool, gripper, and TAP sensor for vegetable cutting. **(B)** Calibration of this end-effector.

Torque Mapping Model: As illustrated in Fig. S30, the end-effector for vegetable cutting is modeled as a system consisting of one rigid link and two springs. Therefore, we can convert from the local sensor signals T_{TAP} to T_{hand} by this model using Eq. (S43). Here, T_{hand} represents the total torque applied on the cutting tool, k_1 denotes the stiffness of the soft pad in the gripper, k_2 is the stiffness of the sensor, L_0 is the distance between the force application point and the sensor, and L_1 is the distance between the center of the sensor and the center of the soft pad. F denotes the external load. Based on Eq. (S43), the mapping coefficient from T_{TAP} to T_{hand} can be derived. Through experimental calibration, this coefficient is determined to be 13.6.

$$\begin{aligned} T_{\mathrm{hand}} &= FL_0 \\ &= K_1L_1\theta + K_2\theta \\ &= K_1L_1T_{\mathrm{TAP}} / K_2 + T_{\mathrm{TAP}} \\ &= (1+K_1L_1 / K_2)\, T_{\mathrm{TAP}} \end{aligned} \quad \text{(S43)}$$

Calibration Protocol: First, we mount the sensor and soft pad onto the end-effector at the robotic arm, then install a frame at the tool's tip to maintain a constant distance between the force application point and the sensor. Next, measure the corresponding distance parameters L_1 and L_0 within the model. Finally, rotate the robotic arm to contact the tool's tip with commercial force sensor (0~20 N). The applied torque T_{hand} is calculated by Eq. (S43) based on the output from the commercial force sensor (F) and the TAP sensor (T_{TAP}) to accomplish the calibration.

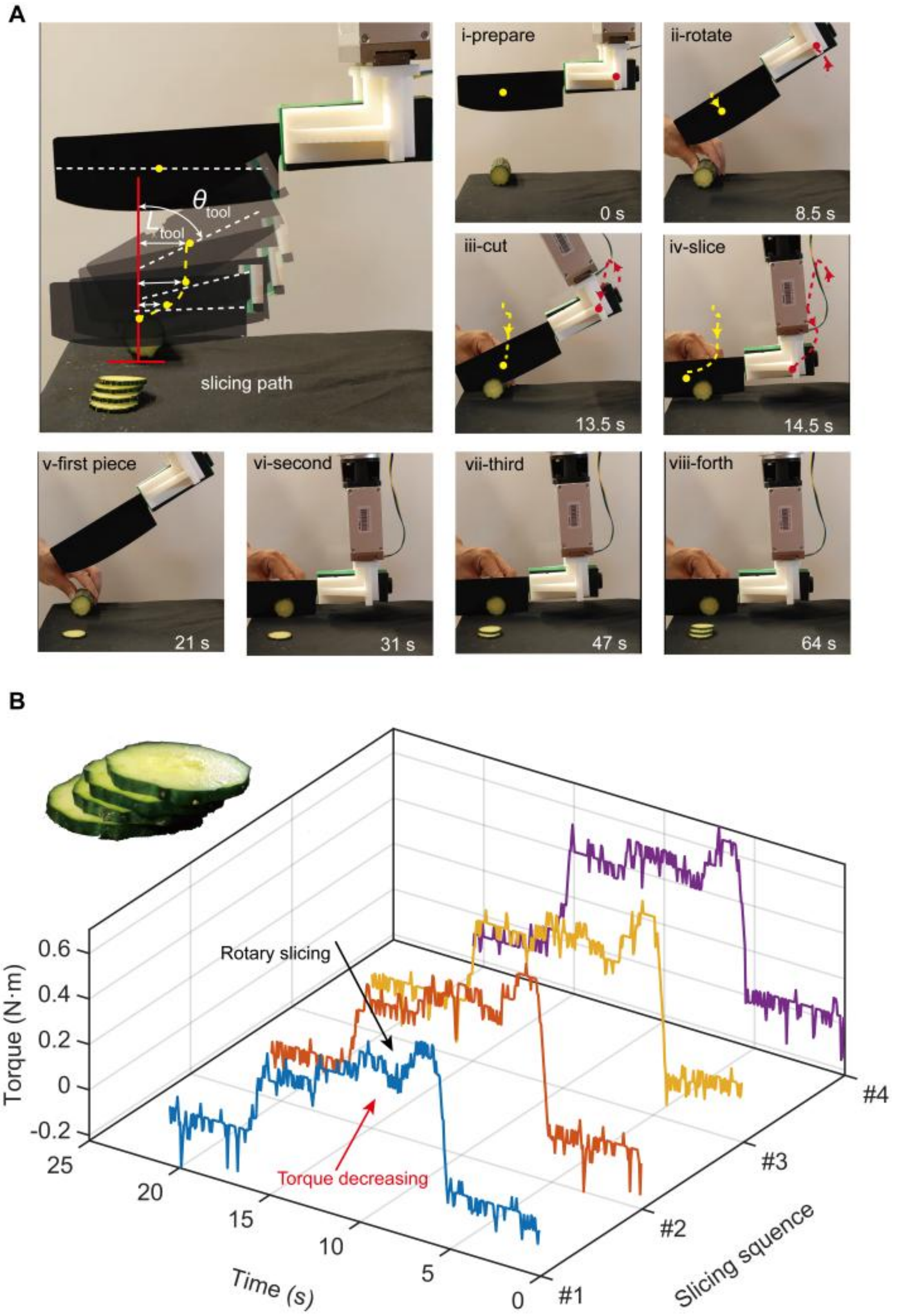

Fig. S31. Slicing vegetables with TAP feedback. (**A**) Illustration of cutting the cucumber into four slices using the rotary slicing method. (**B**) The torque variation is detected by the TAP sensor while slicing the cucumber evenly into four slices.

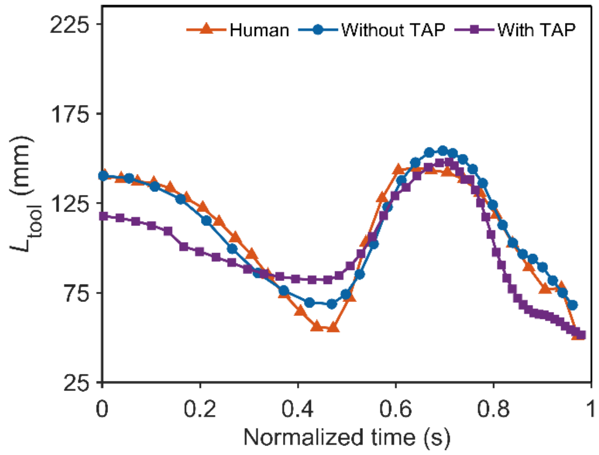

Fig. S32. Variation of tool parameter L_{tool} with different strategies.

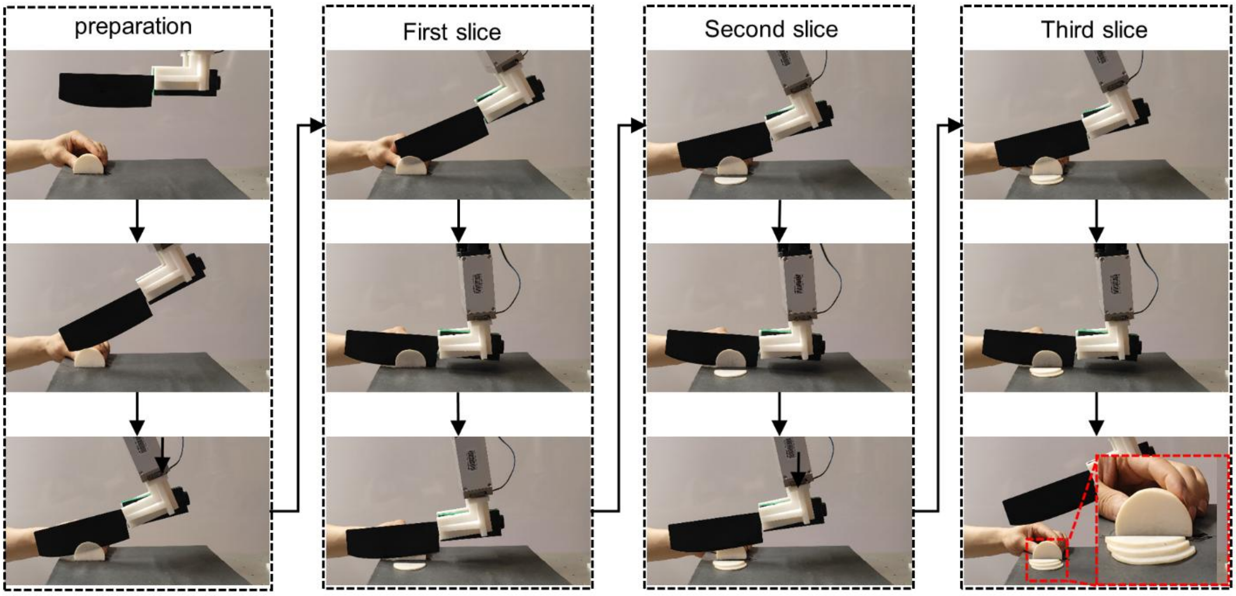

Fig. S33. Schematic diagram of the process of multiple rotary slicing.

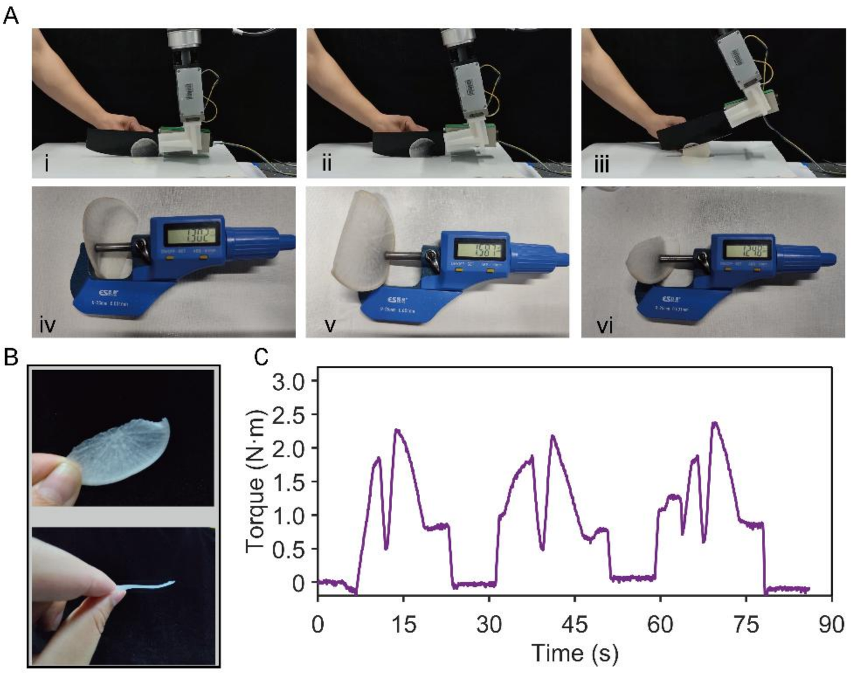

Fig. S34. Rotary slicing for thinner slices. (A) Schematic diagram of slicing daikon. **(B)** Thin slice of daikon. **(C)** The variation of torque during the slicing process.

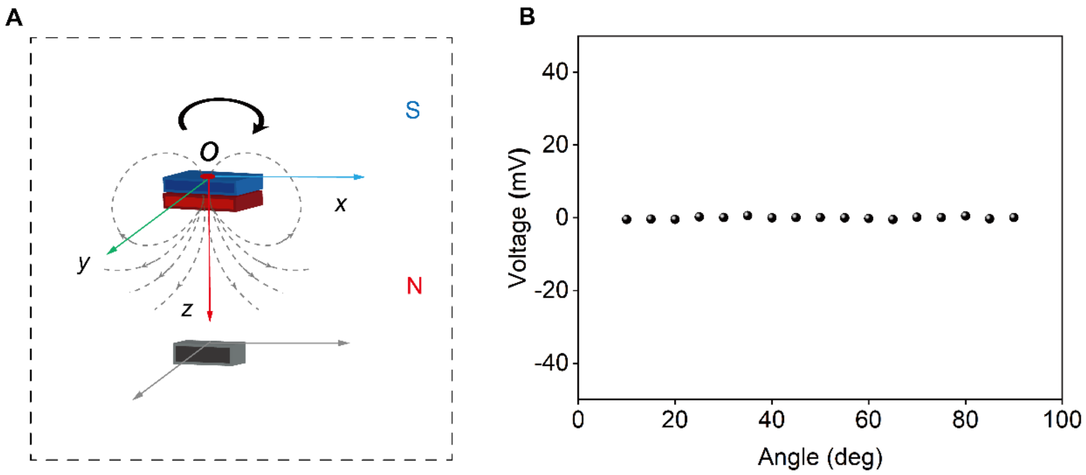

Fig. S35. The output of the Hall element, which is below the center of the magnet during rotation. (**A**) Schematic diagram of the relative motion of the magnet and the Hall element. (**B**) Constant voltage output under torsion.

Supplementary Legends of Movie S1 and S2

Movie S1. Ultra-sensitive torsion angle calibration experiment.

Movie S2. Environment in the loop for precise placement.

Movie S3. Forceful placement of an inverted bottle.

Movie S4. Balance beam stacking challenge: Human vs. Robot.

Movie S5. Slicing daikon.
